# Rapid and accurate determination of atomistic RNA dynamic ensemble models using NMR and structure prediction

**DOI:** 10.1038/s41467-020-19371-y

**Published:** 2020-11-02

**Authors:** Honglue Shi, Atul Rangadurai, Hala Abou Assi, Rohit Roy, David A. Case, Daniel Herschlag, Joseph D. Yesselman, Hashim M. Al-Hashimi

**Affiliations:** 1 Department of Chemistry, Duke University, Durham, NC 27710 USA; 2 Department of Biochemistry, Duke University School of Medicine, Durham, NC 27710 USA; 3 Department of Medicine, Duke University School of Medicine, Durham, NC 27710 USA; 4 Center for Genomic and Computational Biology, Duke University School of Medicine, Durham, NC 27710 USA; 5 Department of Chemistry and Chemical Biology, Rutgers University, Piscataway, NJ 08854 USA; 6 Department of Biochemistry, Stanford University, Stanford, CA 94305 USA; 7 Department of Chemical Engineering, Stanford University, Stanford, CA 94305 USA; 8 ChEM-H Institute, Stanford University, Stanford, CA 94305 USA; 9 Department of Chemistry, University of Nebraska-Lincoln, Lincoln, NE 68588 USA

**Keywords:** RNA, Biophysics, RNA folding, Solution-state NMR

## Abstract

Biomolecules form dynamic ensembles of many inter-converting conformations which are key for understanding how they fold and function. However, determining ensembles is challenging because the information required to specify atomic structures for thousands of conformations far exceeds that of experimental measurements. We addressed this data gap and dramatically simplified and accelerated RNA ensemble determination by using structure prediction tools that leverage the growing database of RNA structures to generate a conformation library. Refinement of this library with NMR residual dipolar couplings provided an atomistic ensemble model for HIV-1 TAR, and the model accuracy was independently supported by comparisons to quantum-mechanical calculations of NMR chemical shifts, comparison to a crystal structure of a substate, and through designed ensemble redistribution via atomic mutagenesis. Applications to TAR bulge variants and more complex tertiary RNAs support the generality of this approach and the potential to make the determination of atomic-resolution RNA ensembles routine.

## Abstract (web summary)

Determining dynamic ensembles of biomolecules is still challenging. Here the authors present an approach for rapid RNA ensemble determination that combines RNA structure prediction tools and NMR residual dipolar coupling data and use it to determine atomistic ensemble models for a variety of RNAs.

## Introduction

The functions of many regulatory RNAs crucially depend on changes in three-dimensional (3D) structures that occur in response to a diverse array of cellular inputs, including the binding of small-molecule ligands^1^, proteins^2^, epitranscriptomic modifications^3^, mutations^4^, and even to nascent RNA elongation during transcription^5^. Initially described as changes from one 3D structure to another, these transitions are now better understood as changes in dynamic ensembles of many inter-converting conformations, from one conformational distribution to another^6^.

To deeply understand RNAs at a level that ultimately makes it possible to rationally manipulate their behavior in drug discovery and synthetic biology, we need the ability to determine their dynamic ensembles at atomic resolution. This however presents a challenge to current biophysical techniques: the information required to specify the position of all atoms in thousands of conformations in an ensemble far exceeds the information content of experimental measurements.

NMR spectroscopy is a rich source of ensemble-averaged measurements, and in combination with computational modeling, has been applied with success to determine ensembles of proteins at atomic resolution^7–9^. In contrast, fewer atomic-level experimental measurements are typically available for characterizing RNA ensembles^6^. Moreover, while computational modeling methods such as molecular dynamics (MD) simulations are needed to address the experimental data gap^10^, nucleic acid force fields remain underdeveloped relative to proteins, and MD simulations of RNAs often poorly predict experimental data even for simple motifs^11,12^. Shortcomings in RNA force field potentials can be addressed using experimental data to readjust the populations of conformers in MD-generated ensembles^13^. However, simulation times are typically short relative to the dynamic timescales measured experimentally, and force field limitations may prevent sampling of particular conformations; ensemble models refined using the experimental data therefore would not include conformations that were not sampled in the original MD simulation. Enhanced sampling approaches can be used, but can lead to an erroneous sampling of high energy conformations, thereby increasing the possibility of over-fitting the data^14^. Compounding these difficulties is the lack of methods for assessing the accuracy of various structural features in an RNA ensemble.

**Fig. 1:**
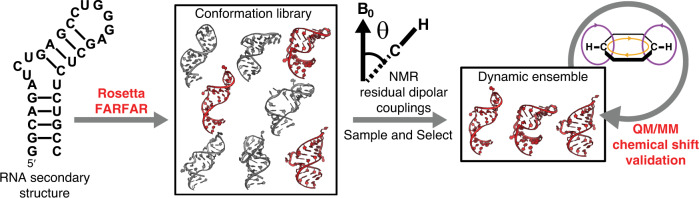
The FARFAR-NMR pipeline. An RNA conformation library is generated by Rosetta FARFAR structure prediction using an RNA secondary structure. The FARFAR-NMR ensemble can be refined from the conformation library using NMR RDCs and cross-validated by computing NMR chemical shifts using QM/MM calculations.

To address the data gap, as well as markedly simplify and accelerate RNA ensemble determination, we took advantage of structure prediction tools that leverage the growing database of RNA structures to directly generate a conformation library that broadly samples energetically favorable 3D conformations given a secondary structure (Fig. 1). We used Fragment Assembly of RNA with Full-Atom Refinement (FARFAR)^15^, given its high performance in extensive tests of blind prediction of 3D RNA structure^16^. We then determined RNA ensembles by using previously published NMR residual dipolar coupling (RDC) data^17,18^ to guide the selection of conformers from the FARFAR-library^12,19,20^. Finally, we used quantum-mechanical calculations of NMR chemical shifts^21^ and cross-validation analysis^12^ to test the accuracy of the generated ensembles (Fig. 1).

## Results

### FARFAR-library better predicts TAR RDCs compared to MD-generated library

**Fig. 2:**
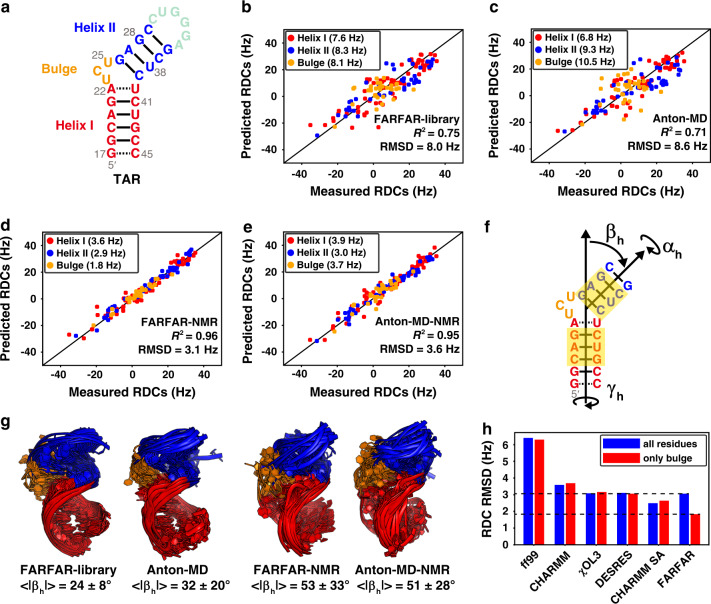
Using FARFAR-NMR to determine a TAR ensemble. **a** Secondary structure of TAR. **b**–**e** Comparison between measured and predicted TAR RDCs for the **b** FARFAR-library (*N* = 10,000), **c** Anton-MD library (*N* = 10,000), **d** FARFAR-NMR (*N* = 20), and **e** Anton-MD-NMR (*N* = 20) ensembles. Values in parentheses denote the RDC RMSD. RDCs are color-coded according to the structural elements in **a**. **f** Definition of the three inter-helical Euler angles (*α*_h_, *β*_h_, *γ*_h_) describing the relative orientation of the two helices (“Methods”). The bps of the upper and lower helices used for defining the Euler angles are highlighted in yellow. **g** Structural overlay of the TAR libraries and NMR refined ensembles (*N* = 20) (“Methods”). |*β*_h_| is the average absolute magnitude of the bend angle. **h** Comparison of RDC RMSD overall (blue) or bulge (red) residues for ensembles generated using different MD force fields (ff99^50^, CHARMM36^26^, ff99bsc0χOL3^51^, and modified ff14 DESRES^55^), multiple simulations using a simulated annealing approach (CHARMM SA), and FARFAR.

We tested our approach on the transactivation response element (TAR) (Fig. 2a) from HIV-1^22,23^, which has served as a model system for bulge motifs. As one of the most common RNA secondary structural elements, bulges often serve as dynamic joints connecting helical elements, enabling their relative orientation to change adaptively during folding and function^12,22–24^. We used FARFAR to directly generate a conformation library (*N* = 10,000) from an input TAR secondary structure only constraining Watson–Crick base pairs (bps) observed by NMR and assuming an idealized A-form geometry^25^ for these bps while predicting the structure for all remaining nucleotides (“Methods”). We then tested and optimized the FARFAR-library using a rich data set of four independent RDCs (~8 RDCs per nucleotide) previously measured for four TAR molecules that were variably elongated to modulate alignment relative to the NMR magnetic field^12,20^.

Intriguingly, the FARFAR-library showed better agreement with the RDCs (Fig. 2b) as compared to a previously reported^12^ TAR library (Anton-MD) that was generated by subjecting an experimentally determined NOE-based NMR structure of TAR (PDBID 1ANR)^22^ to MD simulations with the CHARMM36 force field^26^ (Fig. 2c; RMSD 8.0 versus 8.6 Hz). An optimized ensemble with *N* = 20 conformers (Supplementary Fig. 1b) was generated (“Methods”) by using the agreement with the RDCs to guide selection of conformers from the FARFAR-library (Fig. 2d)^12,19^. The optimized FARFAR-NMR ensemble also better predicted the RDCs (RMSD 3.1 Hz) relative to the optimized Anton-MD-NMR ensemble (RMSD 3.6 Hz) obtained using a similar procedure and the Anton-MD library (Fig. 2e and Supplementary Fig. 1b). Cross validation^12,14^ showed that the improved RDC agreement was not due to over-fitting (Supplementary Fig. 1c, d).

The improved agreement observed with the FARFAR-NMR ensemble, while small, was surprising given that the global inter-helical distribution^27^ of the Anton-MD-NMR TAR ensemble had been independently validated via X-ray scattering interferometry (XSI)^28^. Indeed, the FARFAR-NMR and Anton-MD-NMR ensembles show comparable agreement with the RDCs measured for helical bps (Fig. 2d, e) and the two ensembles sample similar inter-helical orientational distributions (Fig. 2f, g and Supplementary Fig. 2a–d).

Rather, the improved agreement was primarily driven by the RDCs measured in the locally more flexible bulge residues. For the FARFAR-NMR ensemble, the RDC RMSD (1.8 Hz) for bulge residues (Fig. 2d) was within experimental error (~2 Hz), but it was substantially higher (3.7 Hz) for the Anton-MD-NMR ensemble (Fig. 2e). The bulge RDC RMSD could not be improved by running MD simulations using different force fields or by running multiple simulations using the CHARMM36 force field with simulating annealing to more broadly sample conformational space (“Methods”) (Fig. 2h). It appears that improved conformational sampling in the FARFAR-library makes it possible to surpass the accuracy with which the TAR bulge can be described using conventional MD simulations. This improved performance is particularly noteworthy when considering that solving a high-resolution structure and then running MD can in totality consume several months and often years whereas the FARFAR-library is generated within 24 h running on 100 cores in parallel.

### FARFAR-NMR ensembles better predict chemical shifts compared to MD

**Fig. 3:**
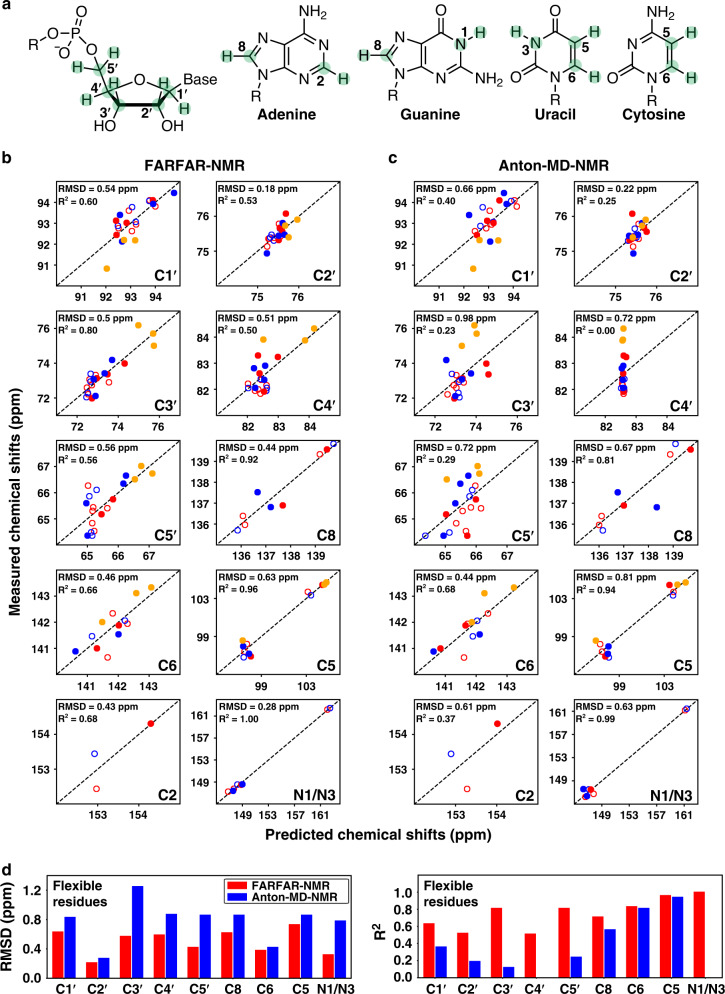
Evaluating TAR ensembles using chemical shifts. **a** Chemical structures of the sugar and base moieties with chemical shift probes used to test ensemble accuracy highlighted in green. **b**, **c** Comparison of measured and predicted ^13^C/^15^N chemical shifts for **b** FARFAR-NMR (*N* = 20) and **c** Anton-MD-NMR (*N* = 20) by AF-QM/MM (“Methods”). Values are color-coded according to the structural elements in Fig. 2a. Chemical shifts for central Watson–Crick bps within A-form helices (C19-G43, A20-U42, G21-C41, A27-U38, G28-C37) are denoted using open circles. A correction was applied to the predicted chemical shifts (“Methods”) as described previously^29^. For ^1^H chemical shifts see Supplementary Fig. 3. **d** Comparison of RMSD (left) and *R*^2^ (right) between measured and predicted ^13^C/^15^N chemical shifts for flexible residues (U23, C24, U25, A22-U40, G26-C39, C29-G36, G18-C44) for FARFAR-NMR (red) and Anton-MD-NMR (blue).

To further evaluate the accuracy of the FARFAR-NMR TAR ensemble, we substantially expanded the breadth and depth of atomic-level experimental data that can be brought to bear when evaluating the accuracy of RNA ensembles by predicting ensemble-averaged ^1^H, ^13^C, and ^15^N chemical shifts (~15 chemical shifts per nucleotide) using quantum-mechanical AF-QM/MM calculations^21,29^. Although rich in structural information that is complimentary to that obtained from RDCs, the agreement between measured chemical shifts and values predicted from crystal structures of nucleic acids has traditionally been poor^21,30^. We recently showed in studies of DNA duplexes that at least some of this disagreement originates from neglecting ensemble-averaging when predicting ^13^C chemical shifts^29^. This revelation and the improved FARFAR-NMR TAR ensemble led us to test this approach on flexible RNAs and to extend it by incorporating ^1^H and ^15^N shifts (Fig. 3a).

Remarkably, good agreement was observed between the measured ^1^H, ^13^C, and ^15^N base and sugar chemical shifts and values back-calculated for the FARFAR-library (Supplementary Fig. 3a). The agreement improved substantially for the FARFAR-NMR ensemble following RDC optimization for 48% of the atom-types analyzed, while the agreement was unaffected for the remaining atom-types (Fig. 3b and Supplementary Fig. 3b). The agreement was poor for any one conformer member of the ensemble, underscoring the critical importance of ensemble-averaging (Supplementary Fig. 4). The *R*^2^ values were >0.5 for ~70% of all the nuclei examined and the RMSD for ^13^C (0.18–0.63 ppm) was decreased relative to the value of 3.6 ppm obtained previously for a static representation of a simple RNA motif^21^ and that obtained for RDC-selected DNA duplex ensembles (0.66–1.27 ppm)^29^ using the same QM/MM approach. The agreement with an independent set of measurements that were not used in ensemble determination and that is highly sensitive to structural features of the base, sugar, and backbone strongly suggests that the FARFAR-NMR ensemble describes the solution behavior of TAR with atomic accuracy (<2 Å, “Methods”).

In sharp contrast, the agreement was much weaker for the Anton-MD-NMR ensemble relative to FARFAR-NMR for 90% of the atom-types, particularly for the sugar chemical shifts, some of which show no apparent correlation even following RDC optimization (Fig. 3c, d, Supplementary Fig. 3c–h and Supplementary Discussion). These results establish the utility of chemical shifts computed using quantum-mechanical calculations in RNA ensemble validation and also show that the improved accuracy of the FARFAR-NMR ensemble relative to Anton-MD-NMR is even greater than revealed by the RDC data alone (also see below).

### FARFAR ensemble broadly samples the sugar-backbone conformation at bulge nucleotides

**Fig. 4:**
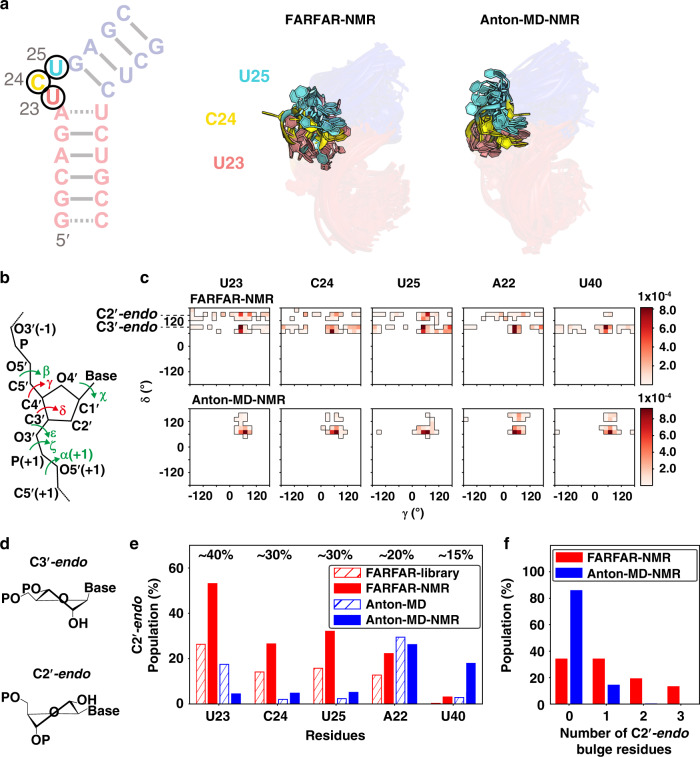
FARFAR-NMR ensemble broadly samples non-canonical sugar-backbone torsion angles relative to Anton-MD-NMR. **a** Overlay of the dynamic ensembles of the TAR bulge. **b** RNA backbone torsion angles exhibiting different and similar distributions between Anton-MD-NMR and FARFAR-NMR are colored red and green, respectively (“Methods”). **c** 2D density maps of *δ* versus *γ* comparing Anton-MD-NMR and FARFAR-NMR ensembles (*N* = 2000) for bulge residues as well as A22 and U40. The bin width is 20°. **d** Structure of the ribose moiety in C3′-*endo* and C2′-*endo* conformations. **e** Population of C2′-*endo* pucker at bulge residues as well as A22 and U40 in the FARFAR-library (*N* = 10,000, red open), FARFAR-NMR (*N* = 20 × 100 = 2000, red fill), Anton-MD library (*N* = 10,000, blue open) and Anton-MD-NMR (*N* = 20 × 100 = 2000, blue fill). Experimental estimates of the C2′-*endo* population based on ^13^C chemical shifts are indicated above the bars (“Methods”). **f** The population of conformers in the ensemble as a function of the number of C2′-*endo* bulge residues for FARFAR-NMR (red, *N* = 20 × 100 = 2000) and Anton-MD-NMR (blue, *N* = 20 × 100 = 2000).

We compared the FARFAR-NMR and Anton-MD-NMR ensembles (Fig. 4a) to better understand the features responsible for the more accurate ensemble description of the TAR bulge. Approximately 75% of the conformers in the FARFAR-NMR ensemble have the canonical UCU bulge, while the remaining 25% have a non-canonical AUC bulge that forms through a single nucleotide register shift (Supplementary Fig. 5a and Supplementary Discussion). In contrast, conformers in the Anton-MD-NMR ensemble sample a broader set of junction topologies some of which deviate from the NMR-derived Watson–Crick pairing (Supplementary Fig. 5b and Supplementary Discussion), potentially contributing to the poor agreement observed for the imino ^15^N/^1^H chemical shifts (Fig. 3c and Supplementary Fig. 3).

In addition, despite having a narrower range of junction topologies, the sugar-pucker distributions (defined by angle *δ*) for bulge residues in the FARFAR-NMR ensemble are broader (Fig. 4b, c), substantially enriched in the non-canonical C2′-*endo* conformation (Fig. 4d) relative to the Anton-MD-NMR ensemble, and are in better agreement with the NMR-derived^31^ sugar puckers (Fig. 4e and Supplementary Fig. 6a). Moreover, ~15% of the conformers in the FARFAR-NMR ensemble had all three bulge residues simultaneously in the C2′-*endo* conformation whereas none did in the Anton-MD-NMR ensemble (Fig. 4f). Excluding all conformers containing C2′-*endo* sugar puckers in U23, C24, U25, A22 or U40 from the FARFAR-library diminished the RDC agreement to a level similar to the Anton-MD derived ensembles again confirming that the improved agreement is not due to over-fitting of the data (Supplementary Fig. 6b, c). Similar behavior was observed for the backbone torsion angle *γ* (Fig. 4b), which shows a greater sampling of non-*gauche* + angles in the FARFAR relative to the Anton-derived ensembles (Fig. 4c and Supplementary Fig. 6b, d). This difference can account for the better agreement observed for sugar chemical shifts^31^ in the FARFAR-NMR versus Anton-MD-NMR ensembles (Fig. 3b–d).

It should be noted that sugar repuckering observed in the FARFAR-NMR ensemble likely occurs on the fast nanosecond to microsecond timescales and is distinct from the slower microsecond to millisecond timescale repuckering modes reported recently for TAR using NMR chemical exchange measurements that are coupled to localized changes in base pairing and secondary structure when forming low-populated “excited states”^31^.

### Atomic view of coaxial stacking and cooperative flipping of bulge nucleotides

**Fig. 5:**
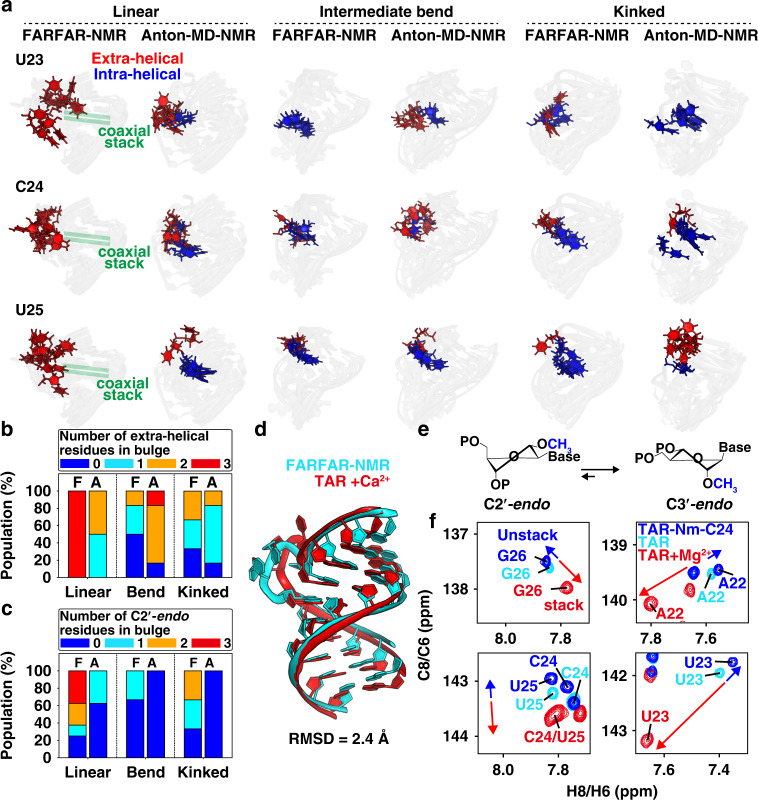
Cooperative extra-helical flipping and sugar repuckering of bulge residues are coupled to coaxial stacking. **a** Overlay of conformers showing motions in bulge residues for linear (|*β*_h_| < 45°), intermediate bend (45° < |*β*_h_| < 70°) and kinked (|*β*_h_| > 70°) inter-helical conformations in the FARFAR-NMR and Anton-MD-NMR ensembles. **b**, **c** The fractional populations of conformers with the number of **b** extra-helical and **c** C2′-*endo* residues (color-coded) in the bulge residues (U23, C24, and U25) as a function of bending angle in the FARFAR-NMR (“F”) and Anton-MD-NMR (“A”) ensembles (*N* = 20). **d** Comparison of one coaxially stacked FARFAR-NMR conformer with the crystal structure of Ca^2+^-bound TAR (PDBID: 397D)^33^. **e** Nm shifts sugar-pucker equilibrium towards C3′-*endo*. **f** Overlay of 2D [^13^C, ^1^H] HSQC NMR spectra of the aromatic spins for TAR-Nm-C24 without Mg^2+^ (blue) with blue arrows indicate unstacking, TAR without Mg^2+^ (cyan) and TAR with 3 mM Mg^2+^ (red) with red arrows indicating increased coaxial stacking.

What causes conformers in the FARFAR-NMR ensemble to more greatly sample non-canonical sugar-backbone conformations at the bulge relative to conformers in the Anton-MD-NMR ensemble? Unpaired pyrimidine RNA nucleotides unconstrained by other interactions are enriched in the C2′-*endo* conformation (C2′-*endo*:C3′-*endo* is 40:60)^32^; C3′-*endo* becomes the predominant sugar-pucker when the nucleotides form bps or stack intra-helically^31^. Consistent with these expectations, in the FARFAR-NMR ensemble, ~80% of the residues with C2′-*endo* sugar puckers were extra-helical (Fig. 5a and Supplementary Fig. 5a). Moreover, linear inter-helical conformers within the FARFAR-NMR ensemble had a strong preference to have all three bulge residues simultaneously flip out and be enriched in C2′-*endo* sugar puckers (Fig. 5b, c, Supplementary Fig. 5a and Supplementary Movie 1). Such coaxial inter-helical conformations have previously been hypothesized to exist within the TAR ensemble based on the Mg^2+^ dependence of the inter-helical ensemble^24^ and a crystal structure of Ca^2+^-bound TAR^33^.

Strikingly, one of the coaxial conformations in the FARFAR-NMR ensemble superimposes with the Ca^2+^-bound TAR crystal structure with a heavy-atom RMSD of 2.4 Å (Fig. 5d). Thus, the TAR crystal structure captures a substate of the ensemble in solution, providing support for the validity of the FARFAR ensemble and underscoring limitations of static structures as accurate representations of RNAs in solution. In sharp contrast, none of the conformations in the Anton-MD-NMR ensemble had the two helices coaxially stacked, and none had all three bulge residues simultaneously flipped out and with C2′-*endo* sugar pucker (Fig. 5a–c and Supplementary Fig. 5b). Based on the population of conformations in which all three bulge residues are simultaneously flipped out relative to the population in which individual bulge residues are flipped out in the FARFAR-NMR ensemble (Fig. 5a and Supplementary Movie 1), the three bulge residues simultaneously flip out with estimated cooperativity of ~2 kcal/mol (“Methods”). This cooperativity may arise because flipping of all three bulge residues permits favorable coaxial stacking of the helices (Fig. 5a, Supplementary Fig. 5a and Supplementary Movie 1). Indeed, changing the sequence identity of the Watson–Crick bps at the interface of the two TAR helices such to promote inter-helical stacking^34^ results in a predominantly coaxially stacked conformation in which all three bulge residues are simultaneously flipped out.

### Further test of the TAR ensemble using atomic mutagenesis

We put key atomic features of the FARFAR-NMR ensemble to a test by rationally redistributing the conformer populations via atomic mutagenesis. The bulge residues in kinked, unstacked conformations are more likely to adopt the C3′-*endo* sugar pucker relative to these residues in the coaxially stacked conformations (Fig. 5c). This difference leads to a prediction: substitutions like 2′-*O*-Methyl (Nm) modifications (Fig. 5e) at U23 or C24 that bias the sugar pucker toward C3′-*endo* will favor the unstacked over stacked conformational states, although the effect will be small (~0.2 kcal/mol per substitution)^35^.

To test this feature of the ensemble, we incorporated Nm modifications at U23 and C24 and assessed the resultant ensemble using NMR chemical shift measurements. Indeed, methylating either U23 or C24 resulted in chemical shift perturbations in 2D NMR spectra of TAR (Fig. 5f and Supplementary Fig. 7) throughout the bulge and neighboring residues that are directed towards the chemical shifts of the unstacked conformation^24^, as expected for a cooperative redistribution in favor of the kinked conformation.

### Applications to TAR bulge variants and in presence of Mg^2+^

**Fig. 6:**
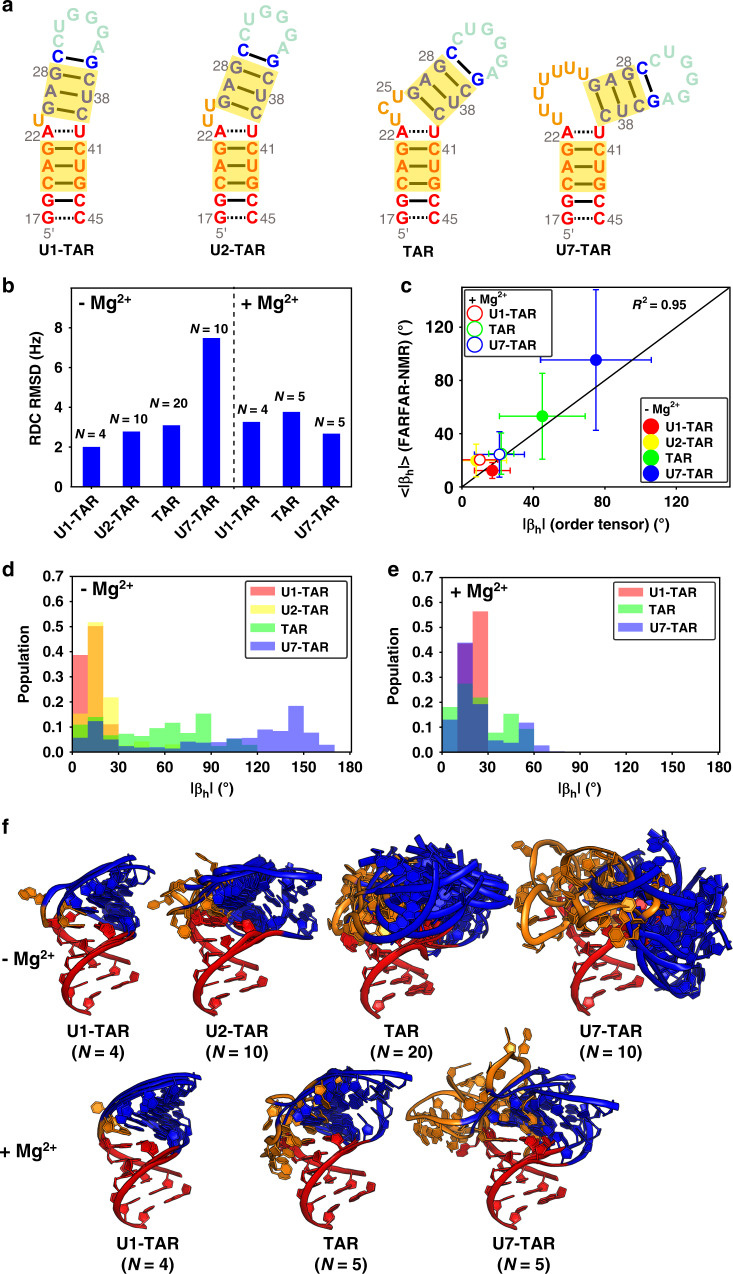
Dynamic ensembles of TAR and its bulge variants in the absence and presence of 3 mM Mg2^+^. **a** Secondary structure of TAR and its bulge variants (U1-TAR, U2-TAR, and U7-TAR). The bps at different helices used for defining the Euler angles are highlighted in yellow. **b** RDC RMSD for ensembles of TAR variants with ensemble size *N* obtained using FARFAR-NMR. **c** Comparison between the average bend angle (<|*β*_h_|>) and its standard deviation for the FARFAR-NMR (*N* = 2000, “Methods”) derived ensembles with the best-fit |*β*_h_| values obtained from an order tensor analysis of the RDCs^24^. The error bar in the order tensor analysis corresponds to half the cone radius angle assuming an isotropic model^69^. **d**, **e** Distributions of the inter-helical bend angle magnitude |*β*_h_| for the ensembles (*N* = 2000, “Methods”) in the absence **d** and presence **e** of Mg^2+^. **f** The FARFAR-NMR ensembles of TAR and its bulge variants in the absence (upper) and presence (lower) of 3 mM Mg^2+^. The ensemble size (*N*) is labeled below for each TAR bulge variant. Motifs in the ensembles are color-coded as in **a**.

To test the generality and limits of our approach, we used FARFAR-NMR to generate ensembles for three additional TAR mutants (Fig. 6a) containing one (U1-TAR), two (U2-TAR), and seven (U7-TAR) bulge nucleotides in the absence and presence of Mg^2+^. The conformational dynamics of these TAR bulge variants have recently been characterized using NMR^24^, though with only a single set of RDCs for each variant, other than for U2-TAR in absence of Mg^2+^ where two sets of RDC measurements were made^20^. With the exception of U7-TAR in the absence of Mg^2+^, the agreement observed between RDCs measured for these TAR mutants and values computed for the FARFAR-library or FARFAR-NMR ensembles was similar to that obtained for TAR (Fig. 6b and Supplementary Fig. 8). The lower agreement for U7-TAR in the absence of Mg^2+^ may reflect limited structural information for kinked RNAs with long bulges^24^ and/or a need to sample a much larger number of conformations.

The FARFAR-NMR ensembles reproduce trends in ensemble properties observed previously for the bulge variants based on an independent analysis of RDCs and chemical shifts^24^. The average and standard deviation of the inter-helical bend angle in the FARFAR-NMR ensembles are in good agreement with values reported based on an order tensor analysis of the RDCs^24^, which does not involve explicit ensemble modeling (Fig. 6c). In addition, the distributions of the bend angle have the expected bimodal character^24^ with a narrow stacked state and broader set of kinked conformations and with Mg^2+^ increasing the population of the stacked state (Fig. 6d–f). These results suggest that FARFAR-NMR can be used to generate ensembles for simple RNA motifs under a variety of conditions in solution.

### Initial applications to other RNAs

**Fig. 7:**
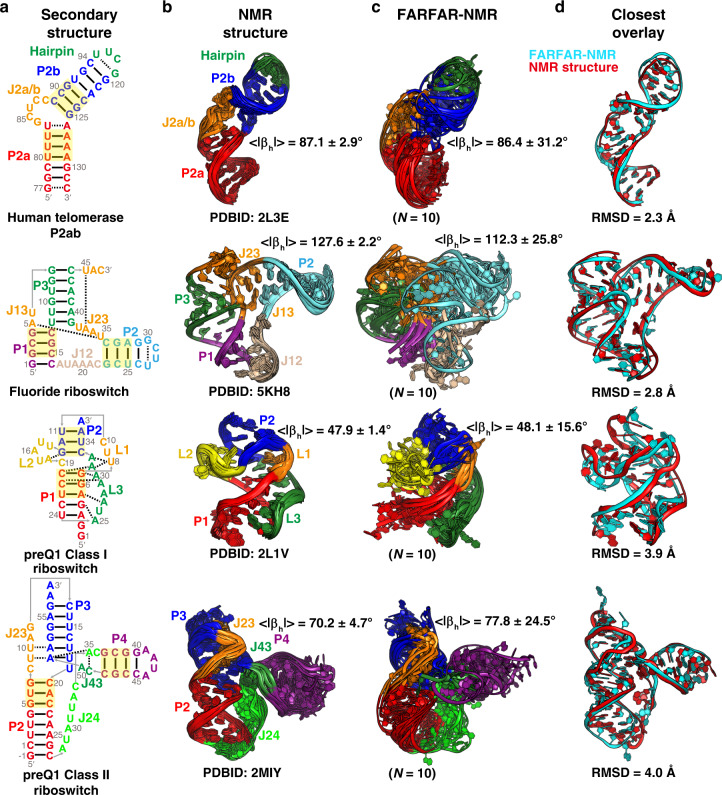
Initial ensembles of various RNA. **a** The RNA secondary structure for each RNA from top to bottom: human telomerase P2ab, fluoride riboswitch, preQ1 Class I riboswitch and preQ1 Class II riboswitch. The bps at different helices used for defining the Euler angles are highlighted in yellow. **b** The NMR structure bundles. **c** The FARFAR-NMR ensembles with ensemble size (*N*) labeled below. Different structural elements are color-coded according to **a**. The average bend angles < |*β*_h_|> (*N* = 10 × 200 = 2000) of the two helices are labeled along with the ensembles. **d** Comparison of the FARFAR-NMR conformer (cyan) with the closest heavy-atom RMSD to the NMR structure (red).

To test the general applicability of FARFAR-NMR to larger and more complex RNAs, we used FARFAR to generate conformation libraries for four additional RNAs: human telomerase P2ab^36^, the fluoride riboswitch apo state^37^, the preQ1 Class I riboswitch holo state^38^ and the preQ1 Class II riboswitch holo state^39^. These RNAs range in size between 35 and 59 nucleotides and include three riboswitches with complex tertiary structures (Fig. 7a). The secondary structures of these RNAs were inferred from prior NMR studies^36–39^. In addition to the secondary structure, long-range non-Watson–Crick bps, which can be identified based on analysis of imino resonances were also specified as restraints during the FARFAR ensemble calculations (“Methods”). We determined preliminary ensembles for these RNAs using a single set of previously published RDCs and compared the results with the reported solution NMR structures determined using a combination of RDCs and NOE data.

In all cases, the FARFAR-NMR ensembles generated using the FARFAR-library reproduced the RDCs (RDC RMSD = 2.7–3.3 Hz) within experimental precision (Supplementary Fig. 9). The ensembles in all cases contain substates that are similar to those in the NMR structures, superimposing with all-atom RMSDs of 2.3–4.0 Å (Fig. 7b–d). The inter-helical orientation in the FARFAR-NMR ensembles also agreed well with that from the NMR structures (Fig. 7b, c). These examples support the generality of the approach but are expected to have limited precision since they were determined based on a single set of RDCs. Further refinement of these ensembles will require measurements of additional RDC data sets followed by evaluation using our chemical shift approach.

## Discussion

FARFAR-NMR lays the foundation for a new paradigm for ensemble determination by combining the most easily obtained and most reliably measured NMR data with 3D structure prediction. The approach can immediately be applied to render many existing NOE-based NMR structures of RNAs currently in the PDB into dynamic ensembles, without the need for additional measurements, and the accuracy of these ensembles can be evaluated using published chemical shift data. It should therefore be possible to rapidly and greatly expand the number of atomistic models of ensembles available for RNAs, and new RNA structures can now be reported as ensembles, not merely as measures of structural uncertainty but as more accurate and complete representations of the RNA’s structural properties.

While we have focused on FAFAR, our approach can be extended to other RNA structure prediction programs^40^ and can also incorporate other sources of experimental data^6^. Given its higher throughput, FARFAR-NMR may enable broader and deeper explorations of ensemble behavior across multiple different RNA motifs under varying solution conditions, providing a broad view of the RNA ensemble landscape that is impossible to attain with current methods. The approach can also be extended to proteins by leveraging advances in protein structure prediction to determining protein ensembles^41^.

Our ability to extensively explore potential conformations of the TAR bulge, and to extensively test the accuracy of resulting ensembles using chemical shifts resulted in unexpected new insights about the TAR ensemble. We have obtained an unprecedented atomic view of the bulge ensemble and how coaxial stacking of helices cooperatively expels bulge residues into an extra-helical conformation with non-canonical sugar-backbone conformations.

Our results show that it is now possible to bring to bear the power of the NMR chemical shift to assess the accuracy of RNA ensembles. Information about motional averaging obtained from accurate ensembles allows accurate chemical shifts to be computed even for highly flexible residues. In the future, it may be possible to directly use chemical shifts in ensemble determination. Such an approach might obviate the need for multiple RDC data sets and further accelerate ensemble determination.

Our results also suggest that there may be biases in MD in at least some current RNA force fields^11^. These force fields favor intra-helical conformations with canonical sugar-backbone conformations for the flexible nucleotides in and around the bulge of TAR. By using chemical shifts and RDCs, it should be possible to greatly expand the data that is available for testing and guiding the optimization of MD-based methods.

While the conformational sampling in the FARFAR-NMR approach is limited by the current repertoire of high-resolution structures of RNA motifs, we can anticipate continual improvements as more structures of RNAs are determined. Future studies should explore other approaches for determining ensembles from the FARFAR generated library including Bayesian approaches^13^ and compare the generated ensembles with those generated using data-driven approaches such as simulated annealing^42^ and restrained MD^43^. Our preliminary application to complex tertiary RNAs underscores the importance of having information regarding long-range contacts when generating the FARFAR-library (Supplementary Fig. 9).

In conclusion, FARFAR-NMR lays the foundation for a new paradigm for RNA ensemble determination by combining reliably measurable NMR data with 3D structure prediction. The approach can immediately be applied to render many existing NMR structures of RNAs that were determined using conventional approaches into dynamic ensembles, and their accuracies can be tested using the available chemical shift data. The approach is general, rapid, and can also incorporate additional sources of experimental data. Given its ease of implementation and higher throughput, FARFAR-NMR has the potential to unleash the ensemble description of RNAs to all corners of biology, from which a deeper and broader understanding of folding and function will undoubtedly emerge.

## Methods

### Generating ensembles using FARFAR

FARFAR^15^ is implemented as the *rna_denovo* program in Rosetta Software Suite. FARFAR requires as input the RNA sequence, which can be constrained by an optional secondary structure. The input secondary structures for TAR and its bulge variants were derived based on the imino ^1^H resonances in NMR spectra. Therefore, Watson–Crick base pairing was imposed for G18-C44, C19-G43, A20-U42, G21-C41, G26-C39, A27-U38, G28-C37, and C29-G36, whereas nucleotides in the apical loop and bulge, and the terminal G17-C45 bp were unconstrained. Given the indirect NMR observation of the A22-U40 bp in U2-TAR by H6(C5)NN experiment as reported previously^24^, two sets of FARFAR simulations were conducted for each TAR variant with A22-U40 either paired or unpaired. Interestingly, with the exception of U2-TAR, constraining A22-U40 when generating FARFAR-libraries did not improve the agreement with the RDCs for TAR and other variants. This highlights the importance of differentiating between well-formed versus labile bps, an important advantage offered by NMR as compared to other methods for determining secondary structure such as chemical probing. Thus, all the FARFAR-libraries in this study correspond to those in which the A22-U40 bp is not constrained, except for the U2-TAR ensemble in the absence of Mg^2+^. The input secondary structures of TAR and its bulge variants were summarized in Supplementary Tables 2–5.

The secondary structure constraints of other RNAs including human telomerase P2ab^36^, fluoride^37^, and preQ1 Class I^38^ and Class II^39^ riboswitches were derived from prior NMR studies based on a combination of observed imino ^1^H resonances, HNN-COSY experiments, direct observation of resonances in slow exchange, and ^15^N chemical shifts, and are summarized in Supplementary Tables 6–9. For these RNAs, non-Watson–Crick base pairing was also imposed during the FARFAR calculations using the “-obligate_pair_explicit” flag with the specification of Leontis–Westhof (LW) classification (Supplementary Tables 6–9). Specifically, for the U-G bp in UUCG apical loop, the bp geometry was set to “tSW” and only G in the *syn* conformation (*χ* between 0 and 100°)^44^ was kept. Additional inspection of steric clashes was performed on these bps specified by the “-obligate_pair_explicit” flag to remove stereochemically unreasonable structures. To significantly reduce the run time, we modeled the helices of TAR and its bulge variants, human telomerase P2ab, and fluoride riboswitch as static idealized A-form helices shown previously to satisfy NMR RDC data in RNA^24,25^. For preQ1 Class I and II riboswitches, since the helices are involved in tertiary interactions, a better RDC RMSD was achieved while allowing the helices to deviate from the idealized A-form during the calculation.

*rna_helix.py* is a python wrapper for the Rosetta executable *rna_helix*. *rna_helix.py* is available in $ROSETTA/tools/rna_tools/bin, where $ROSETTA is the Rosetta installation path.

*rna_helix.py -seq gcag cugc -resnum 18-21 41-44 -o helix1.pdb*

*rna_helix.py -seq gagc gcuc -resnum 26-29 36-39 -o helix2.pdb*

in which RNA sequences of both strands as well as their residue indices are required as input. While generating FARFAR-library assuming idealized A-form helices, we then executed *rna_denovo* with the following command:

*rna_denovo -nstruct 100 -s helix_1.pdb helix_2.pdb -fasta input.fasta -secstruct_file input.secstruct -minimize_rna true*

where *-nstruct* is the number of models per run, which is 100 in the current study, *-fasta* is the path of a fasta file containing the RNA sequence, *-secstruct* is the path of a file (input.secstruct) containing the RNA secondary structure in dot-bracket notation, *-minimize_rna true* minimizes the RNA after fragment assembly, and *-s* specifies the path to the pdb files that contain static structures of our helices we do not wish to generate via fragment assembly to save computation (the fasta, secstruct files as well as example commands can be found in Supplementary Tables 2–9).

The entire procedure was repeated 100–200 times and 10,000 structures were randomly selected from the entire resulting output with Rosetta energy units < 0 (for TAR and its bulge variants) to remove models that potentially may have chain breaks and severe steric clashes, which do not satisfy the RDCs (Supplementary Fig. 1a), to generate the FARFAR-library (*N* = 10,000). The Rosetta energy cutoff for human telomerase P2ab, fluoride riboswitch, preQ1 Class I riboswitch and preQ1 Class II riboswitch was set to be 50, 200, 150, 100, respectively. The cutoff was determined as described for TAR (Supplementary Fig. 1a).

### Molecular dynamics (MD) simulations

Simulations of HIV-1 TAR starting from the PDB structure 1ANR using the CHARMM36 force field^26^ (8.3 μs) were performed as described previously^12^. The structures from this simulation were then clustered based on the heavy-atom RMSD of TAR (excluding the apical loop and terminal bp) using the *cluster* command in the CPPTRAJ suite^45^ to give 16 structures, which served as starting points for the simulated annealing runs. The simulated annealing simulations using the CHARMMM 36 force field were performed with the GROMACS MD simulation package^46^. GROMACS force field files for CHARMM36 were obtained from (http://mackerell.umaryland.edu/charmm_ff.shtml#gromacs) as of March 2019. The structures were solvated with a rhombic dodecahedral box of TIP3P^47^ water molecules, with box size chosen such that the boundary was at least 10 Å away from any of the RNA atoms, and was then neutralized using Na^+^ ions. After energy minimization without restraints, the system was then gradually heated to a temperature of 300 K using a Nose–Hoover thermostat^48^ (*τ* = 0.1 ps), under constant volume conditions for 100 ps with harmonic restraints on the solute (20 kJ/mol/nm^2^). The restraints were gradually reduced in two 50 ps NVT equilibration steps (10 and 5 kJ/mol/nm^2^, respectively). This was followed by two 50 ps NVT and NPT equilibration steps at 300 K without restraints on the solute. NPT equilibration was performed using a modified Berendsen thermostat with a stochastic term^49^ with *τ* = 0.1 ps and at 1 bar using a Berendsen barostat with *τ* = 2.0 ps. Simulated annealing was then performed by subjecting the system to a pair of successive 50 ps NVT and NPT equilibration steps at 400 and 300 K. This was followed by a 5 ns NVT equilibration step at 300 K prior to the production run (500 ns). A non-bonded cutoff of 10 Å was used for treating short-range non-bonded interactions while the particle mesh Ewald method was used to treat long-range electrostatic interactions. Non-bonded van-der Waals forces were switched to 0 from 8 to 10 Å. Covalent bonds involving hydrogen were constrained using the LINCS algorithm to enable the use of a 2 fs timestep. A total of 625 equally spaced snapshots were obtained from each of the 16 production runs to get create a pool of 10,000 conformers for RDC selection.

MD simulations of TAR starting from the PDB structure 1ANR using the ff99 force field^50^ with and without *χ*_OL3_ corrections for RNA^51^ were performed using periodic boundary conditions as implemented in the AMBER MD simulation package^52^. All starting structures were solvated using a truncated octahedral box of TIP3P^47^ water molecules with box size chosen such that the boundary was at least 11 Å away from any of the RNA atoms for all simulations with the ff99 force field with *χ*_OL3_ corrections. For the TAR simulations using the ff99 force field without *χ*_OL3_ corrections, the system was solvated in a truncated octahedral box of SPC/E water molecules such that the boundary was at least 15.4 Å away from any of the RNA atoms. Na^+^ ions treated using the Joung–Cheatham parameters^53^ was then added to neutralize the charge of the system in all cases. The system was then energy minimized in two stages with the solute being fixed (with restraint of 500 kcal/mol/Å^2^) during the first stage. Equilibration and production runs (1 μs) were then performed as described previously^54^.

Simulations of TAR starting from PDB 1ANR using the DESRES force field^55^ were performed using the GROMACS MD simulation package^46^. DESRES force field files for GROMACS were obtained from a port by Giovanni Bussi (https://github.com/srnas/ff/tree/desres). The starting structure (PDB 1ANR) was solvated using a rhombic dodecahedral box of TIP4P-D^56^ water molecules, with box size chosen such that the boundary was at least 10 Å away from any of the RNA atoms. Na^+^ ions treated using the parameters from MacKerell et al. ^57^ were then added to neutralize the charge of the system. After energy minimization without restraints, the system was then gradually heated to a temperature of to 298 K using a modified Berendsen thermostat with a stochastic term^49^ (*τ* = 0.1 ps), under constant volume conditions for 100 ps with harmonic restraints on the solute (1000 kJ/mol/nm^2^). The system was then allowed to equilibrate for 100 ps under constant pressure (1 bar), using the Parinello–Rahman barostat^58^ (*τ* = 2 ps) and temperature (at 298 K, using a modified Berensen thermostat^49^, *τ* = 0.1 ps) conditions, with harmonic restraints on the solute (1000 kJ/mol/nm^2^). This was followed by NPT equilibration for 30 ns without harmonic restraints, following by a production run of 1 μs. A non-bonded cutoff of 9 Å was used for treating short-range non-bonded interactions while the particle mesh Ewald method was used to treat long-range electrostatic interactions. All bonds were constrained using the LINCS algorithm to enable the use of a 2 fs timestep. A set of evenly (5 ps) spaced snapshots were used for subsequent analysis of all simulations using the CPPTRAJ suite of programs^45^.

Additional MD simulations using the ff99 force field with *χ*_OL3_ corrections were also performed to assess the extent to which sampling of sugar puckers in the bulge could be influenced by changes in the starting structures used for the simulations (Supplementary Fig. 10). Two starting structures were derived from PDB 1ANR with the sugar puckers of U23 (TAR^U23 C2′-*endo*^) and U25 (TAR^U25 C2′-*endo*^) individually switched from C3′-*endo* to C2′-*endo*, and another was a conformer from the FARFAR-NMR ensemble (TAR^FARFAR^) in which the sugar puckers of U23, C24 and U25 were all C2′-*endo*. The starting structures for the TAR^U23 C2′-*endo*^ and TAR^U25 C2′-*endo*^ simulations were generated by the superposition of a C2′-*endo* uridine nucleotide onto U23/U25 in 1ANR using the uridine base atoms, and replacing the sugar-backbone atoms with those of the superimposed C2′-*endo* uridine. For TAR^U23 C2′-*endo*^ and TAR^U25 C2′-*endo*^, only the backbone of the C2′-*endo* uridine was fixed during an energy minimization, while for TAR^FARFAR^, all the heavy atoms were fixed during minimization.

### NMR residual dipolar coupling (RDC) data

The RDC data (sugar C1′-H1′/C2′-H2′/C3′-H3′/C4′-H4′ and base C8-H8/C6-H6/C2-H2/C5-H5/N1-H1/N3-H3) used in the RNA ensemble determination were reported previously^12,36–39^ and are summarized in Supplementary Table 1. The raw data can also be downloaded from https://github.com/alhashimilab/RDC.

### RDC calculations

Ensemble-averaged RDCs were calculated by computing the RDCs for each conformer in an ensemble using the program PALES^59^. PALES computes RDCs based on global molecular shape. The RDC was computed using a cylindrical wall model using the following command:

*pales -pdb input.pdb -inD input_rdc.tab -outD output_rdc.tab -H -pf1 -wv 0.022*

where *-pdb* is the path of an input PDB file (input.pdb), *-inD* is the path of an input data file indicating the bond vectors for which RDC should be computed (input_rdc.tab), *-outD* is the path of the output file containing all the calculated RDCs, *-H* means selecting all atoms including proton and *-pf1 -wv* specifies the pf1 effective concentration (0.022 g/mL) assuming a rod liquid crystal model.

The RDC values were then averaged over all conformers in an ensemble assuming that they are equiprobable. Individual scaling factors were applied to the predicted RDCs of each construct to account for the difference of alignment magnitude in part arising due to differences in phage concentrations in the experiments, as described previously^12^. The elongated constructs were elongated in silico using an idealized A-form geometry prior to RDC calculation as described previously^12^.

The TAR apical loop was modeled using the wild-type CUGGGA loop^12^. As reported previously for the Anton-MD-NMR ensemble^12^, replacing the loop with the UUCG loop^44^ used to measure RDCs minimally impacted the RDC agreement for the FARFAR ensembles (Supplementary Fig. 1e, f). This is consistent with prior NMR studies showing that the apical loop replacement minimally impacts the dynamics of the bulge^12,60^.

### Sample and select (SAS)

We used the SAS approach^19^ to generate ensembles from a structured pool that best satisfied the measured RDCs. Briefly, a simulated annealing Monte Carlo sampling scheme was used to select an ensemble that minimizes the cost function depicting the differences between the measured and predicted RDCs:

1 $$\chi ^2 = \frac{{\mathop {\sum }\nolimits_j \mathop {\sum }\nolimits_i^N (L_j \times D_{i,j}^{\mathrm{calc}} - D_{i,j}^{\mathrm{exp}})}}{N},$$

$D_{i,j}^{\mathrm{calc}}$ and $D_{i,j}^{\mathrm{exp}}$ are the calculated and measured RDCs, respectively, of the *i*th bond vector measured on the *j*th TAR construct, $L_j$ is the overall scaling factor of alignment magnitude for construct *j*, and *N* is the total number of bond vectors. The initial effective temperature for simulation annealing was 100 and decreased by a factor of 0.9 in every step for a total of 5 × 10^5^ steps. A series of SAS runs were performed varying the ensemble size from *N* = 1 to an ensemble size in which the RDC RMSD reaches a plateau (Supplementary Figs. 1b, 8, and 9). The resultant ensemble size for different RNA ensembles were: *N* = 4 (U1-TAR, no Mg^2+^), *N* = 10 (U2-TAR, no Mg^2+^), *N* = 20 (TAR, no Mg^2+^), *N* = 10 (U7-TAR, no Mg^2+^), *N* = 4 (U1-TAR, with Mg^2+^), *N* = 5 (TAR, with Mg^2+^), *N* = 5 (U7-TAR, with Mg^2+^), *N* = 10 (human telomerase P2ab), *N* = 10 (fluoride riboswitch), *N* = 10 (preQ1 Class I riboswitch) and *N* = 10 (preQ1 Class II riboswitch). To analyze distributions of structural parameters (Figs. 4, 6, 7 and Supplementary Fig. 2), we also generated larger sized ensembles by running SAS multiple times to ensure the total size *N* = 2000 for all systems.

The SAS analysis of TAR and its bulge variants excluded RDCs from the flexible terminal G17-C45 bp as well as those of the C29-G36 bp flanking the apical loop, given differences between the apical loop sequences used to measure RDCs (wild-type or UUCG) and to model (wild-type) TAR. Note that RDCs from G17-C45 and C29-G36 bp were included in the prior study^12^ and this explains the small differences in RDC RMSD for the two Anton-MD-derived ensembles relative to that reported earlier.

### Cross-validation analysis

Cross-validation was performed using two approaches as described previously^12^. In one approach (inactive random, Supplementary Figs. 1c, d, 8, 9), 10% of the RDC data was randomly removed and SAS was used to generate an ensemble. The RMSD between measured and predicted RDCs was then computed for the left out RDC data. This procedure was repeated 10 times and the final RDC RMSD was averaged over all ten independent runs. For TAR (absence of Mg^2+^) where we have RDCs measured on four constructs, a second mode (inactive media, Supplementary Fig. 1c, d) of cross-validation was also performed, in which the RDC data set of each construct was left out individually before running SAS. The RMSD between measured and predicted RDCs was then computed for the left out RDC data set. The final RDC RMSD is averaged over the four iterations corresponding to leaving out each RDC data set.

### NMR chemical shift data

The ^1^H, ^13^C, and ^15^N chemical shift assignments of TAR have been published previously^31,60^ and were compared to quantum-mechanical chemical shift predictions. The numerical populations for C2′-*endo* shown in Fig. 4e were obtained based on the C1′ chemical shift assuming a linear dependence between the range 86 ppm (100 % C2′-*endo*)^54^ and 94 ppm (100 % C3′-*endo*)^31^.

### Automated fragmentation quantum mechanics/molecular mechanics (AF-QM/MM) chemical shift calculations

Chemical shift calculations were performed using a previously described fragmentation procedure^21^. Each RNA structure was subjected to five steps of conjugate gradient minimization with harmonic restraints of 2 kcal/mol Å^2^ on all heavy atoms; this regularizes bond lengths and angles to minimize the noise in the results that can arise from very small changes in these geometric parameters. Next, each structure was broken into “quantum” fragments centered on each nucleotide, containing 2–6 neighboring nucleotides, using a heavy-atom distance cutoff of 3.4 Å. The effects of RNA atoms outside the quantum region, and of water and ions in the solvent, were represented as point charges uniformly distributed on the molecular surface of the quantum region and resolved by fitting to Poisson−Boltzmann calculations using the “solinprot” program from the MEAD package^61,62^. The quantum region was assigned a local dielectric ε of 1 (vacuum); the remaining RNA region had an ε of 4, and the solvent region an ε of 80. GIAO chemical shift calculations were carried out for each fragment, using version 5.0 of the demon-2k program^63^ using the OLYP functional^64^ with the pcSseg-1 (triple-ς plus polarization) basis set optimized for chemical shifts^65^ for the central nucleotide (whose results are reported here), and a DZVP basis for the remaining atoms. Reference shieldings were computed for tetramethylsilane (TMS) using the same functional and basis set. The ensembles of Anton-MD-NMR, FARFAR-NMR as well as a randomly selected Anton-MD and FARFAR-library of size *N* = 20 were examined. A linear correction was applied to the predicted chemical shifts for each nucleus type individually as described previously^29^. Note that R^2^ can be artificially low for spins with small ranges of chemical shifts (e.g., ~1 ppm for C3′/C4′/C6 for central Watson–Crick bps).

### Ensemble analysis

All the ensemble structure visualization was performed in PyMOL (https://pymol.org/). The local backbone and sugar torsion angles (Fig. 4b) were calculated using X3DNA-DSSR^66^. The inter-helical Euler angles (*α*_h_, *β*_h_, *γ*_h_) were computed as described previously^27^ (Figs. 2, 5, 6, 7 and Supplementary Figs. 2, 5). Briefly, the two RNA helices connected by a junction were aligned to two idealized A-form RNA helices (upper helix and lower helix), respectively, and the relative orientation was specified by the inter-helical Euler angles between the upper and lower helix. For TAR and its bulge variants, helix II from G26 to G28 was aligned to the upper helix and helix I from C19 to G21 was aligned to the lower helix, respectively. For human telomerase P2ab, the P2b helix from C89 to G91 was aligned to the upper helix and the P2a helix from U80 to U82 was aligned to the lower helix, respectively. For fluoride riboswitch, the P2 helix from G23 to U25 was aligned to the upper helix and the P1 helix from G2 to G4 was aligned to the lower helix, respectively. For preQ1 Class I riboswitch, the P2 helix from C33 to A35 was aligned to the upper helix and the P1 helix from A5 to G7 was aligned to the lower helix, respectively. For the preQ1 Class II riboswitch, the P4 helix from G37 to G39 was aligned to the upper helix and the P2 helix from G5 to G7 was aligned to the lower helix, respectively. The sign of *α*_h_ and *γ*_h_ is inverted relative to previously reported values^12^ such that a positive and negative inter-helical twist angle (*α*_h_ + *γ*_h_) corresponds to over- and under-twisting, respectively^67^.

Junctional topology (Supplementary Fig. 5) was defined as the base-pairing mode, which is detected by X3DNA-DSSR^66^. If two bases are forming a bp with Leontis–Westhof (LW) classification as “cWW” (e.g., Watson–Crick bp, Wobble bp), a solid line is indicated between the two bases, whereas other LW classifications are indicated as a dashed line (Supplementary Fig. 5).

TAR conformers were considered to be coaxially stacked when the bases comprising A22-U40 and G26-C39 or G21-C41 and U25-U40, were stacked with each other, as defined by X3DNA-DSSR^66^.

The lower bound estimate of pairwise RMSD between two ensembles was defined as the following: Consider two ensembles A and B with size *N*_A_ and *N*_B_. For every conformer in ensemble A, we found the corresponding conformer in ensemble B that has the lowest pairwise RMSD. This procedure was repeated for every conformer in A to obtain a total of *N*_A_ RMSD values. We then took the root mean square of all these *N*_A_ RMSD values. The procedure was also repeated considering ensemble B. Then the minimum of the two root mean square values were selected as a lower bound of the similarity between the two ensembles. By this definition, Anton-MD-NMR and FARFAR-NMR ensembles differ by 3.7 Å while ensembles obtained from multiple independent FARFAR-NMR runs typically differ by 1.4 Å on average.

The cooperativity of flipping bulge nucleotides out was computed from the FARFAR ensemble as follows—for U23, C24, and U25, the probability of independently flipping out was computed as:

2 $$P(nt\,out) = \frac{{N(nt\,out|other\,nt\,in)}}{{N(nt\,out|other\,nt\,in) + N(nt\,in|other\,nt\,in)}},$$

where *N(nt out | other nt in)* is the number of FARFAR-NMR conformers with the nucleotide (nt) of interest being flipped out with the other nucleotides (among U23, C24, and U25) being flipped in, and *N(nt in | other nt in)* is the number of FARFAR-NMR conformers with U23, C24, and U25 flipped in. Flipping in and flipping out of U23, C24, and U25 were gauged by visual examination of the conformers (Supplementary Fig. 5). The probability of simultaneously flipping out U23, C24, and U25 without cooperativity as then computed as the product of the probabilities of independently flipping out each nucleotide as defined above, i.e.,

3 $$P(U23,C24,U25\,out) = P(U23\,out) * P(C24\,out) * P(U25\,out).$$

This was then compared to the observed probability of U23, C24, and U25 being flipped out *P(U23, C24, U25 out obs)*, which was computed as the fraction of FARFAR-NMR conformers with U23, C24 and U25 flipped out (8/20). Cooperativity was defined as:

4 $$- RT\,{\mathrm{ln}}(\frac{{P(U23,C23,U25\,out\,obs)}}{{P(U23,C23,U25\,out)}}),$$

where *R* is the universal gas constant, *T* is the temperature in Kelvin (298 K).

### NMR sample preparation

TAR, TAR-Nm-U23, and TAR-Nm-C24 RNA samples were synthesized using a MerMade 6 Oligo Synthesizer (BioAutomation) via solid-phase synthesis using standard phosphoramidite chemistry and deprotection protocols. 2′-TBDMS protected phosphoramidites (ChemGenes) and 1 μmol standard synthesis columns (1000 Å) were used. The final 5′-DMT (4,4′-dimethoxytrityl) was removed during the synthesis for DMT-off deprotection and PAGE purification. Removal of nucleobase and phosphate protecting groups and cleavage from the 1 μmol columns was achieved using 1 ml of 30% ammonium hydroxide and 30% methylamine (1:1) followed by a 2-h incubation at room temperature. The solution was then air-dried and dissolved in 100 μL DMSO and 125 μL TEA-3HF, followed by 2.5 h incubation at 65 °C following Glen Research protocols (https://www.glenresearch.com/reports/gr19-22) for 2′-*O* deprotection. The sample was then ethanol precipitated overnight, air-dried, then dissolved in water for gel purification using a 20% (w/v) polyacrylamide gel with 8 M urea and 1× Tris/borate/EDTA. The RNA was removed from the excised gel by electro-elution in 1× Tris/acetic acid/EDTA followed by ethanol precipitation. The RNA was annealed in the water at a concentration of 50 µM by heating at 95 °C for 5 min followed by cooling on ice for 60 min. It was then buffer exchanged using an Amicon Ultra-15 centrifugal filter (EMD Millipore) with a 3 kDa cutoff into NMR buffer (15 mM sodium phosphate, 25 mM NaCl, 0.1 mM EDTA) at pH 6.4. The final concentrations were: ~0.8 mM for TAR, 2.5 mM TAR-Nm-U23, and 1.4 mM TAR-Nm-C24. TAR was also buffer exchanged into NMR buffer containing 3 mM Mg^2+^.

### NMR spectroscopy

All the NMR 2D HSQC experiments in this study were carried out on Bruker Avance III 600-MHz NMR spectrometer equipped with a triple-resonance cryogenic probed at 25 °C. NMR Data were processed using NMRpipe^68^ and analyzed using SPARKY (T.D. Goddard and D.G. Kneller, SPARKY 3, University of California, San Francisco), respectively. The resonance assignments for Nm-modified TAR were obtained based on the previously reported assignments of TAR^31,60^ and further confirmed using 2D NOESY experiments.

### Reporting summary

Further information on research design is available in the Nature Research Reporting Summary linked to this article.

## Supplementary information

Supplementary Information

Peer Review File

Reporting Summary

Description of Additional Supplementary Files

Supplementary Movie 1

## Data Availability

The data that support this study are available from the corresponding authors upon reasonable request. Rosetta FARFAR commands as well as its required input files are included in Supplementary Information. The raw RDC data used in this study can be downloaded from https://github.com/alhashimilab/RDC. The FARFAR-NMR and Anton-MD-NMR ensembles of TAR (*N* = 20) as well as the idealized A-form helix used for in silico elongation can be downloaded from https://github.com/alhashimilab/Ensemble. The FARFAR-NMR ensemble of TAR (*N* = 20) with the chemical shifts and RDCs data has been deposited to the Biological Magnetic Resonance Data Bank under the accession code 30788 and the Protein Data Bank under the accession code 7JU1.

## Code availability

The Rosetta software suite is available at https://www.rosettacommons.org/software/academic. The AFNMR programs are available at https://github.com/dacase/afnmr. Custom in-house Python scripts for sample and selection are available at https://github.com/alhashimilab/PySAS. Custom in-house Python scrips for calculation of inter-helical Euler angles are available at https://github.com/alhashimilab/ABG_calc.
