# Supplementary material for: Rapid and accurate determination of atomistic RNA dynamic ensemble models using NMR and structure prediction
Source: Nat Commun. 2020 Nov 2;11:5531. doi: 10.1038/s41467-020-19371-y (PMC7608651; doi:10.1038/s41467-020-19371-y)
Supplement: Supplementary file 1 — Supplementary Information [file 41467_2020_19371_MOESM1_ESM.pdf]

Supplementary Information

Rapid and accurate determination of atomistic RNA dynamic ensemble models using NMR and structure prediction

Honglue Shi[1], Atul Rangadurai[2], Hala Abou Assi[2,3], Rohit Roy[4], David A. Case[5]*, Daniel Herschlag[6,7,8]*, Joseph D. Yesselman[9]*, Hashim M. Al-Hashimi[1,2]*

[1]Department of Chemistry, Duke University, Durham, NC, 27710, USA

[2]Department of Biochemistry, Duke University School of Medicine, Durham, NC, 27710, USA

[3]Department of Medicine, Duke University School of Medicine, Durham NC, 27710, USA

[4]Center for Genomic and Computational Biology, Duke University School of Medicine, Durham, NC, 27710, USA

[5]Department of Chemistry and Chemical Biology, Rutgers University, Piscataway, NJ, 08854, USA

[6]Department of Biochemistry, Stanford University, Stanford, CA, 94305, USA

[7]Department of Chemical Engineering, Stanford University, Stanford, CA, 94305, USA

[8]ChEM-H Institute, Stanford University, Stanford, CA, 94305, USA

[9]Department of Chemistry, University of Nebraska-Lincoln, Lincoln, NE, 68588, USA

Correspondence to: hashim.al.hashimi@duke.edu, jyesselm@unl.edu, herschla@stanford.edu, david.case@rutgers.edu

Supplementary Figures

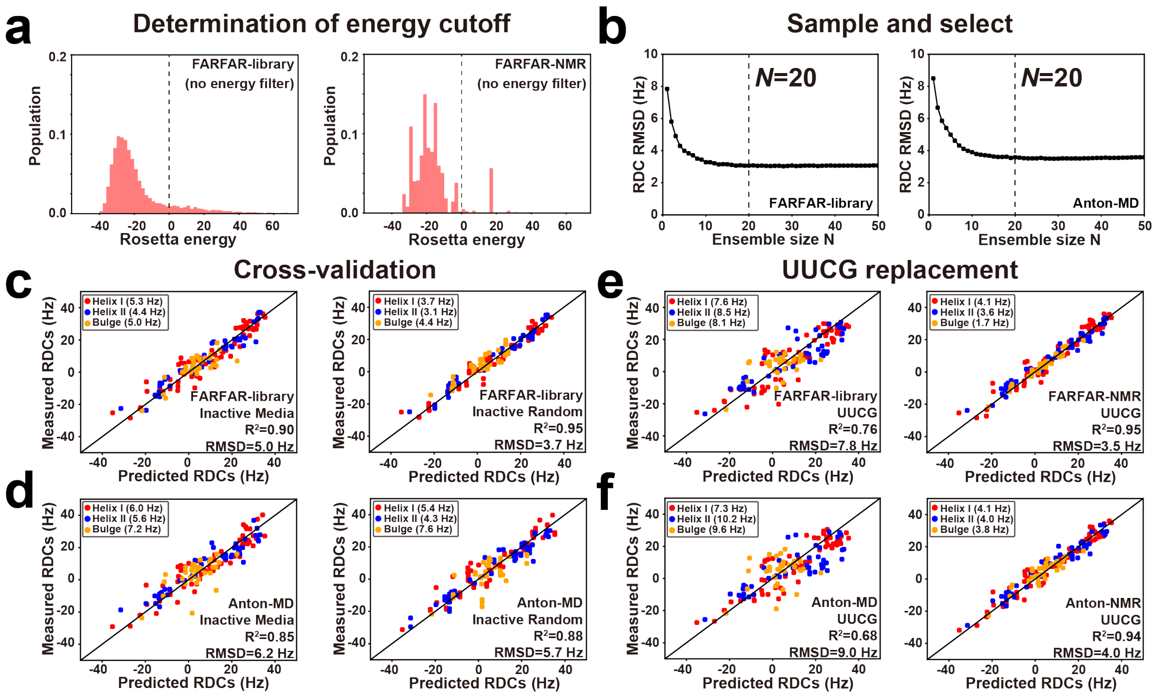

Supplementary Fig. 1 Optimizing TAR ensembles using SAS. (a) Determination of Rosetta energy cutoff. (left) Shown are the distribution of energies of a FARFAR-library (no energy filter) (N = 10,000) generated without pre-filtering based on Rosetta energy (i.e., not excluding structures with Rosetta energy > 0). (right) Distribution of no pre-filtering FARFAR-library in (left) after RDC selection (N = 2,000). The Rosetta energy = 0 is indicated using a dashed line. As structures with energy > 0 were predominantly excluded following SAS, only structures with energy < 0 were retained while generating the FARFAR-library in the main manuscript. (b) RDC RMSD as a function of ensemble size (N) during SAS for (left) the FARFAR-library and (right) Anton-MD. The chosen ensemble size N = 20 is indicated using a vertical dashed line. (c-d) Cross-validation analysis of TAR ensembles. Shown are comparison of measured and predicted RDCs obtained from cross-validation analysis on TAR ensembles using two modes: Inactive Media (left) and Inactive Random (right)(Methods) for (c) the FARFAR-library and (d) Anton-MD. (e-f) Generation of TAR ensembles using UUCG apical loop models[1].

Comparison between measured and predicted RDCs for starting pools with N = 10,000 (left) and comparison between measured and predicted RDCs after SAS with N = 20 (right) from (e) FARFAR-library replacing wild-type CUGGGA loop with a UUCG loop and (f) Anton-MD replacing wild-type CUGGGA loop with a UUCG loop. Replacing the loop with the UUCG loop used to measure RDCs minimally impacted the RDC agreement for both FARFAR and Anton ensembles.

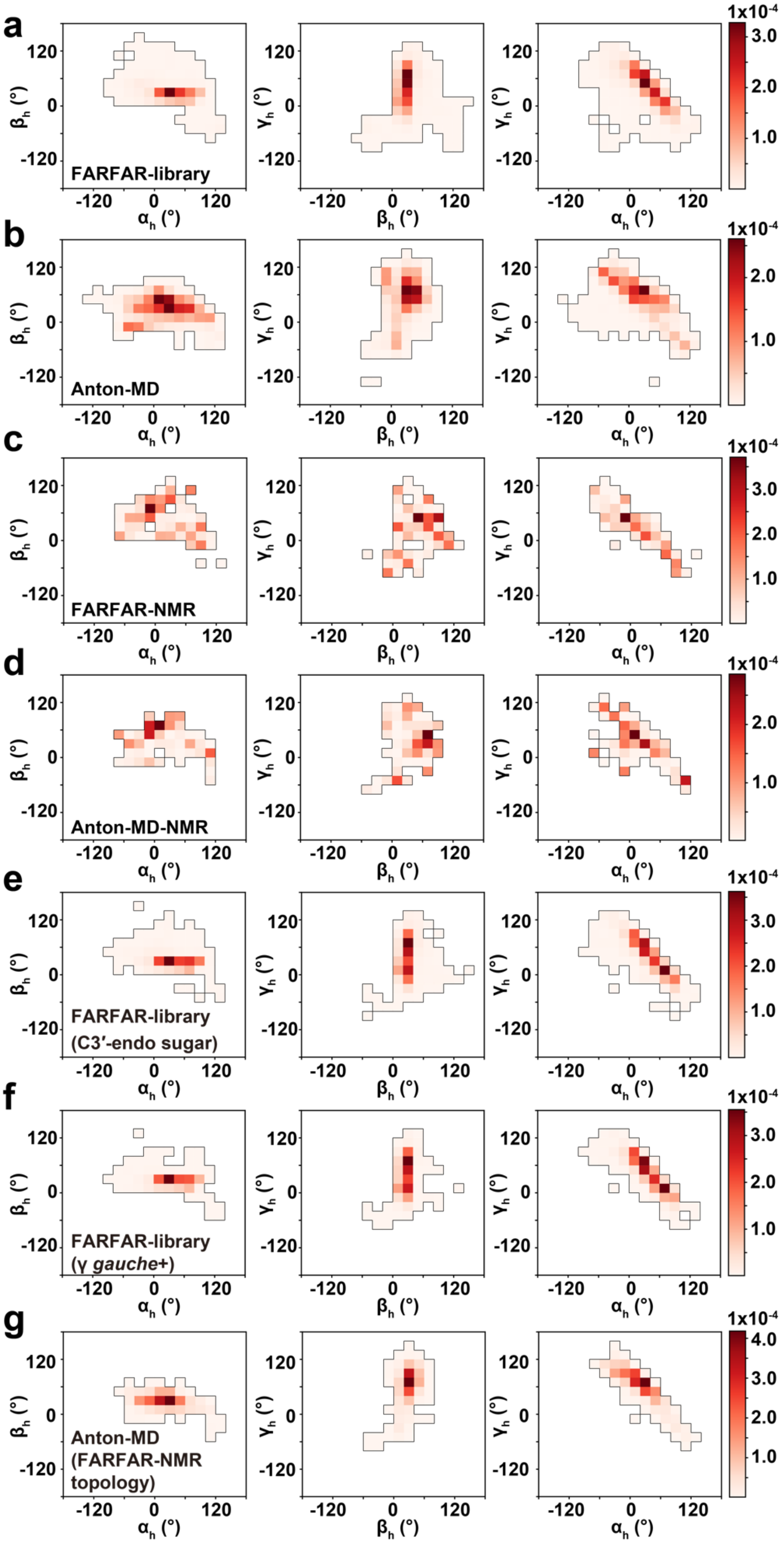
a
b
c
d
e
f
g
FARFAR-library
Anton-MD
FARFAR-NMR
Anton-MD-NMR
FARFAR-library
(C3′-endo sugar)
FARFAR-library
(γ gauche+)
Anton-MD
(FARFAR-NMR
topology)
αh (°)
βh (°)
γh (°)
1x10-4
-120
0
120

Supplementary Fig. 2 Inter-helical orientational distributions of FARFAR and Anton libraries and ensembles. The 2D density map of inter-helical Euler angle (α_h, β_h, γ_h) for (a) FARFAR-library (N = 10,000), (b) Anton-MD (N = 10,000), (c) FARFAR-NMR (N = 2,000), (d) Anton-MD-NMR (N = 2,000), (e-f) two subsets of FARFAR-library: (e) one with U23, C24, U25, A22 and U40 constrained to be C3′-*endo* (N = 4,422), and (f) the other one with U23, C24, U25, A22 and U40 constrained to be only *gauche*+ γ (20°<γ<100°) (N = 3,145), (g) a subset of Anton-MD with conformers retaining the same junction topology as that in FARFAR-NMR (Methods) (N = 2,074). The color-scale for density is given on the right. In all cases, the bin width is 20°.

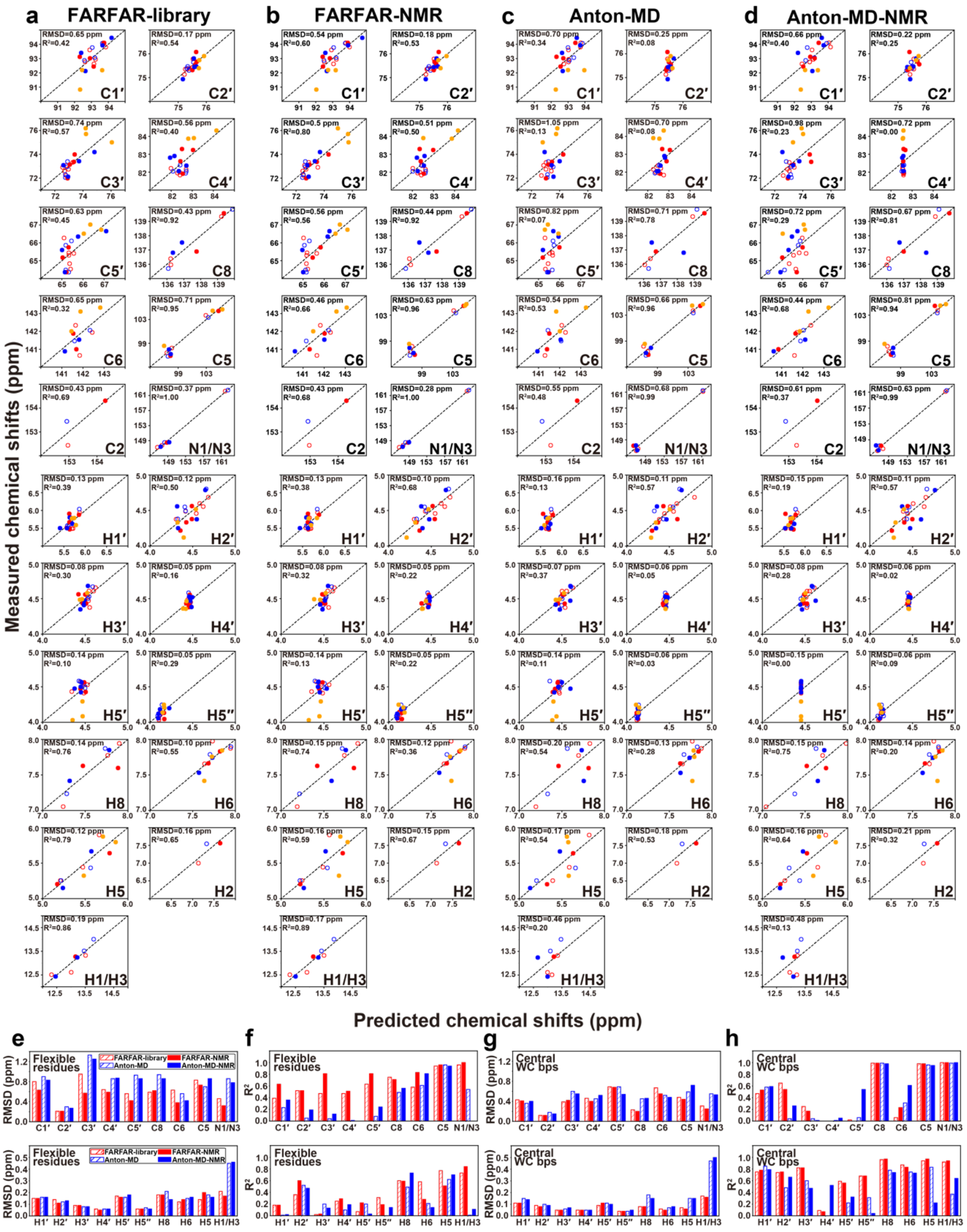

Supplementary Fig. 3 Evaluation of TAR libraries and ensembles via ^{13}C, ^{15}N and ^{1}H chemical shifts. (a-d) Comparison of measured and predicted ^{13}C, ^{15}N and ^{1}H chemical shifts for (a) FARFAR-library, (b) the FARFAR-NMR ensemble, (c) Anton-MD, and (d)

the Anton-MD-NMR ensemble (N = 20 in all cases) by AF-QM/MM (Methods). Chemical shifts are color-coded according to the different structural elements (Fig. 2a). Chemical shifts for the central Watson-Crick bps within A-form helices (C19-G43, A20-U42, G21-C41, A27-U38, G28-C37) are denoted using open circles. A correction was applied to the predicted chemical shifts (Methods) as described previously[2]. (e-h) Comparison of RMSD and R^2 between measured and predicted $^{13}C/^{15}N$ (top) and ^{1}H (below) chemical shifts for (e-f) flexible residues (U23, C24, U25, A22-U40, G26-C39, C29-G36, G18-C44) and (g-h) central Watson-Crick bps for the FARFAR-library (red, open), FARFAR-NMR ensemble (N = 20) (red, fill), Anton-MD (blue, open) and the Anton-MD-NMR ensemble (N = 20) (blue, fill).

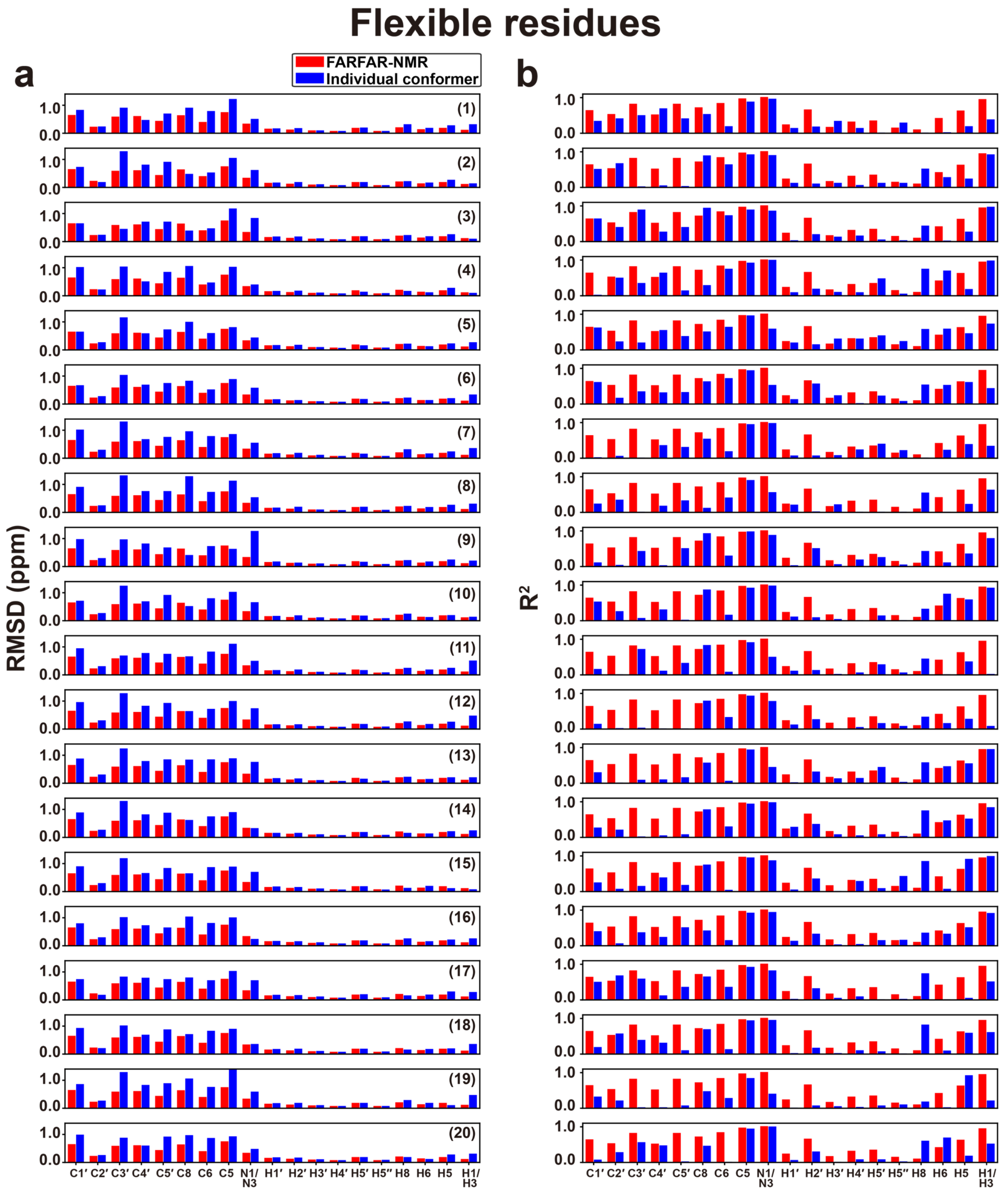

Supplementary Fig. 4 Comparison of agreement of measured and predicted chemical shifts between FARFAR-NMR ensemble and individual conformers. Bar plot of (a) RMSD and (b) R^2 between measured and predicted chemical shifts for FARFAR-NMR ensemble (red) and its individual conformer (blue) for only flexible residues (U23, C24,

U25, A22-U40, G26-C39, C29-G36, G18-C44). All the conformers in FARFAR-NMR ensemble ($N = 20$) are sorted in increasing order of the bend angle magnitude $|\beta_h|$.

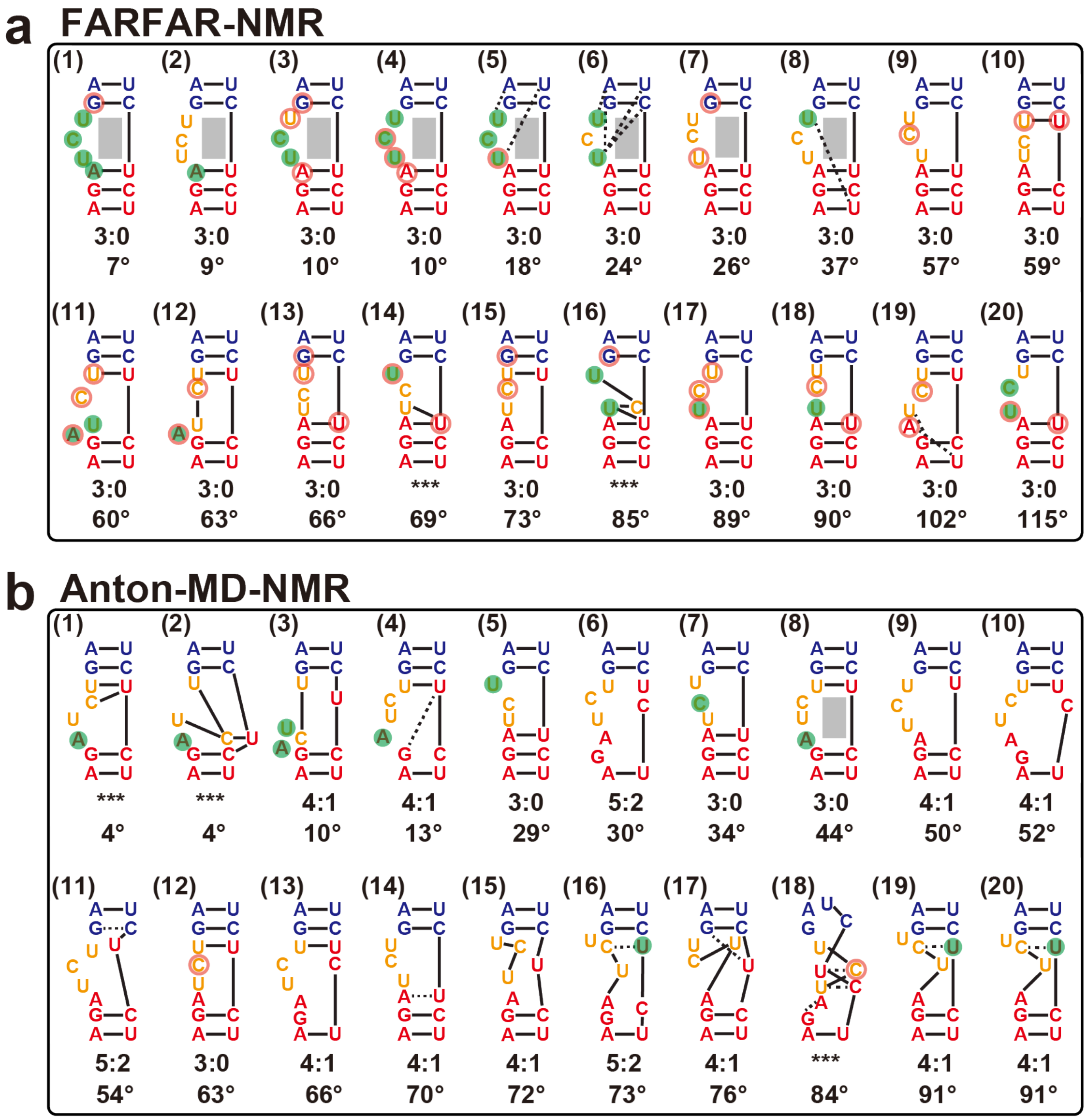

Supplementary Fig. 5 Junction topology scheme of the TAR ensembles. Junction topology in the (a) FARFAR-NMR ($N = 20$) and (b) Anton-MD-NMR ($N = 20$) ensembles. Conformers in each ensemble are sorted in increasing order of the bend angle magnitude $|\beta_h|$, and the junction topology (Methods) as well as the $|\beta_h|$ are labeled below each conformer. Junctional residues (bulge/A22-U40/G26-C39) with C2′-*endo* sugar pucker and non-gauche+ γ (falling outside 20-100°) torsion angle are highlighted with green filled circle and orange open circle, respectively.

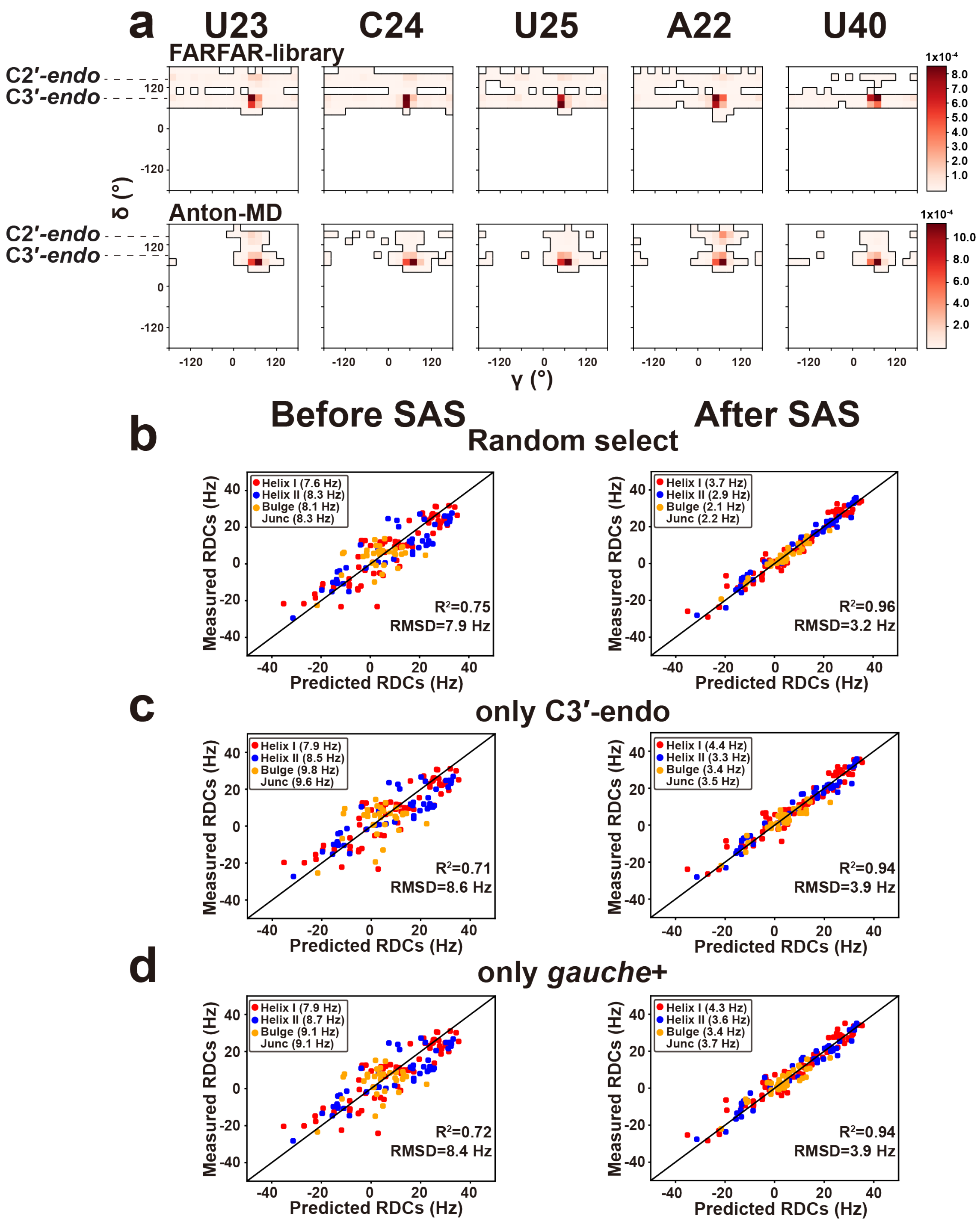

Supplementary Fig. 6 Testing the sensitivity of FARFAR-library and FARFAR-NMR ensembles to variations in local torsion angles. (a) Distribution of backbone torsion angles of the FARFAR-library and Anton-MD. 2D density map of δ versus γ of the bulge residues as well as A22 and U40, for the FARFAR-library and Anton-MD (N = 10,000).

The bin width is 20° for all density maps. (b-d) Comparison of measured and predicted RDCs for a subset of the FARFAR-library (left, $N = 3{,}000$) and ensembles (right, $N = 20$) following SAS on these subset libraries. (b) Randomly selected subset from the FARFAR-library (c) a subset of the FARFAR-library with the sugar puckers of U23, C24, U25, A22 and U40 chosen to be C3′-*endo* (d) a subset of the FARFAR-library with the γ torsion angles of U23, C24, U25, A22 and U40 chosen to be gauche+ (20°<γ<100°). "Junc" denotes bulge residues as well as A22 and U40.

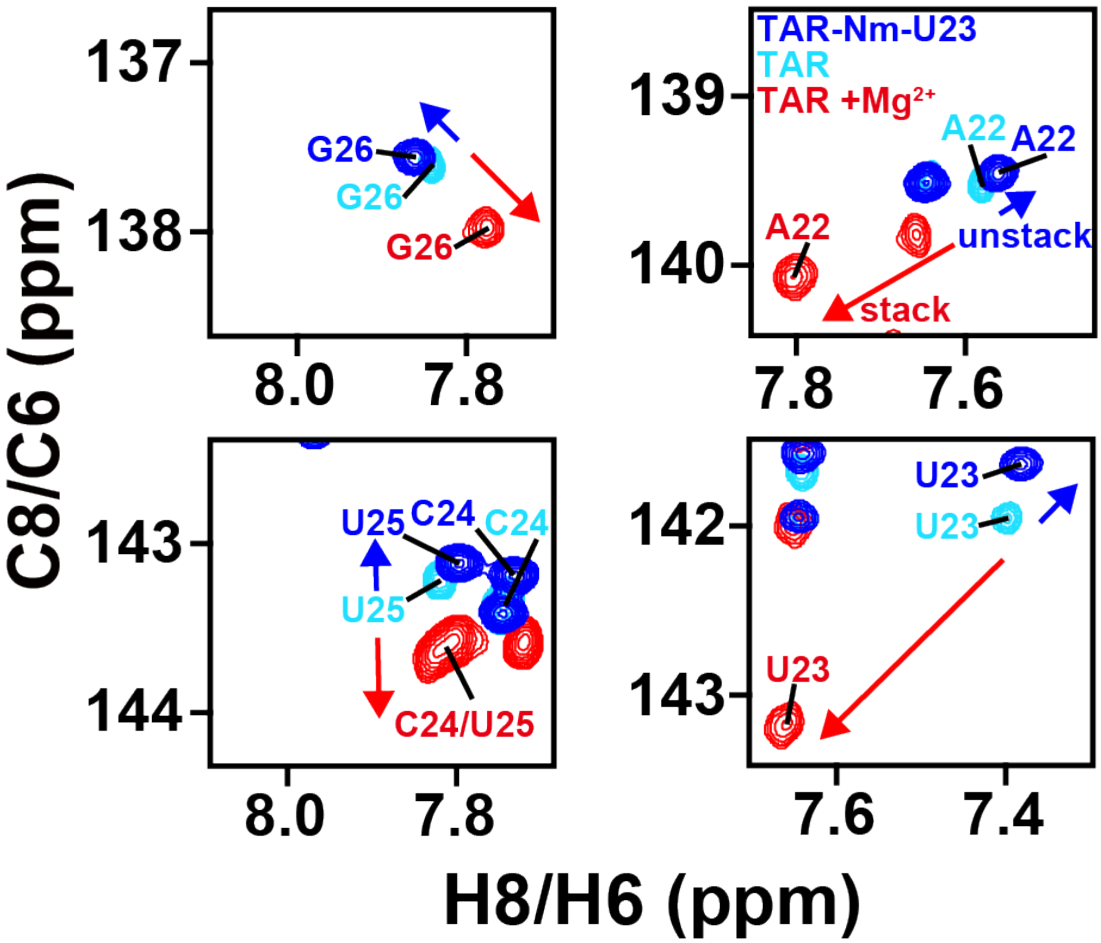

Supplementary Fig. 7 An additional Nm modified TAR showing Nm inducing unstack of TAR. Overlay of 2D [^{13}C, ^{1}H] HSQC NMR spectra of the aromatic region of TAR-Nm-U23 without Mg^{2+} (blue, inducing unstacking). TAR without Mg^{2+} (cyan), and TAR $+Mg^{2+}$ (red, inducing stacking).

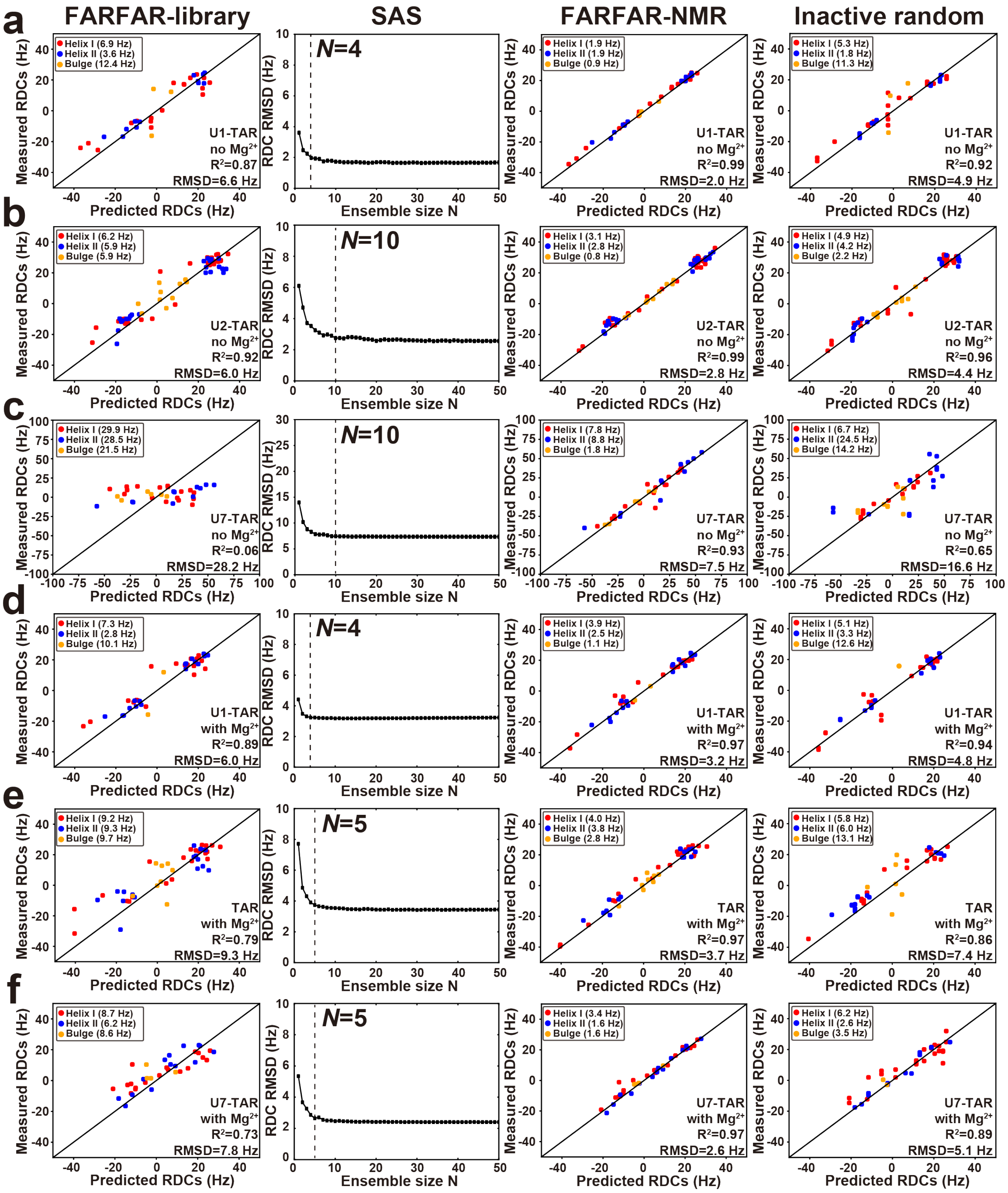

Supplementary Fig. 8 Optimizing ensembles of TAR and its variants. From left to right, comparison of measured and predicted RDCs for the FARFAR-library (N = 10,000), RDC RMSD as a function of ensemble size during SAS (ensemble size N chosen for the final ensembles is indicated as a vertical dashed line), comparison between the measured and predicted RDCs for the FARFAR-NMR ensembles after SAS, and inactive random

cross-validation for (a) U1-TAR no Mg^{2+} (b) U2-TAR no Mg^{2+} (c) U7-TAR no Mg^{2+} (d) U1-TAR with Mg^{2+} (e) TAR with Mg^{2+} and (f) U7-TAR with Mg^{2+}. RDC values are color coded according to structural element as defined in Fig. 2a and Fig. 6a.

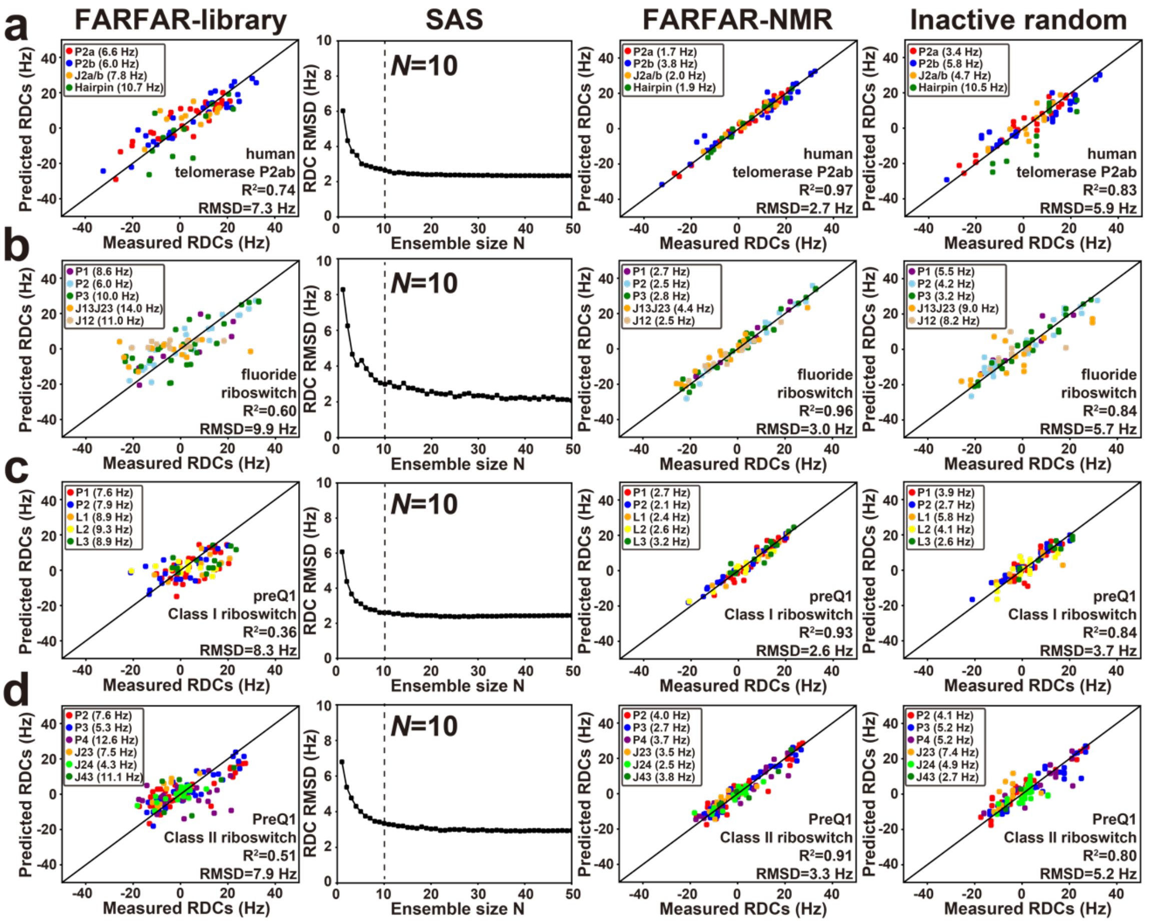

Supplementary Fig. 9 Optimizing ensembles of other RNAs. From left to right, comparison of measured and predicted RDCs for the FARFAR-library (N = 10,000), RDC RMSD as a function of ensemble size during SAS (ensemble size N chosen for the final ensembles is indicated as a vertical dashed line), comparison between the measured and predicted RDCs for the FARFAR-NMR ensembles after SAS, and inactive random cross-validation for (a) human telomerase P2ab (b) fluoride riboswitch (c) preQ1 Class I riboswitch (d) preQ1 Class II riboswitch. RDC values are color coded according to structural element as defined in Fig. 7a.

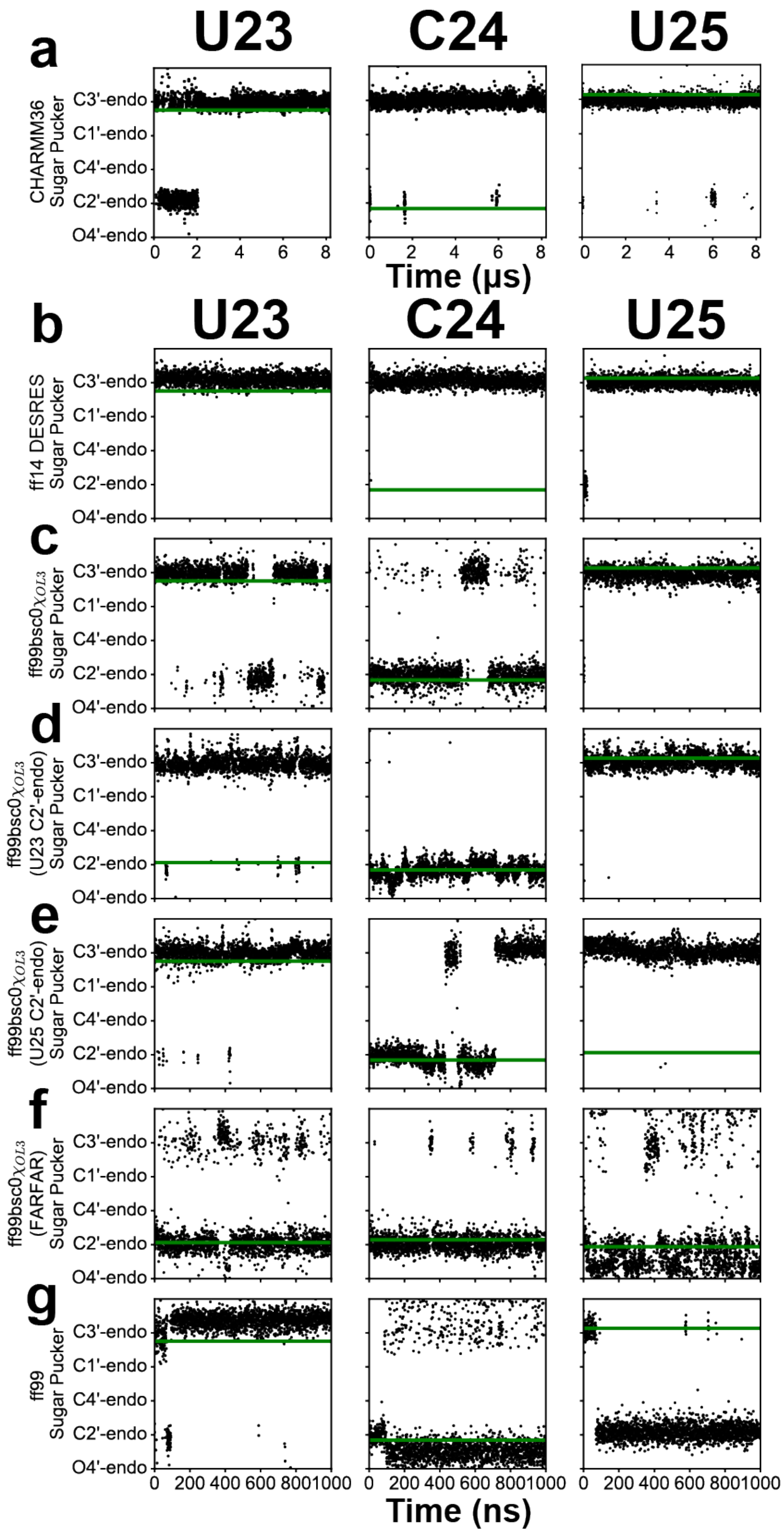

a
U23
C24
U25
CHARMM36 Sugar Pucker
C3'-endo
C1'-endo
C4'-endo
C2'-endo
O4'-endo
0 2 4 6 8
Time (μs)
b
U23
C24
U25
ff14 DESRES Sugar Pucker
c
ff99bsc0χOL3 Sugar Pucker
d
ff99bsc0χOL3 (U23 C2'-endo) Sugar Pucker
e
ff99bsc0χOL3 (U25 C2'-endo) Sugar Pucker
f
ff99bsc0χOL3 (FARFAR) Sugar Pucker
g
ff99 Sugar Pucker
0 200 400 600 800 1000
Time (ns)

Supplementary Fig. 10 Sugar puckers of bulge nucleotides during the course of MD simulations with different force fields and starting structures. The variation of the sugar puckers of U23, C24 and U25 of TAR during the course of (a) an 8.2 μs trajectory using the Anton CHARMM36 force field and (b-g) a series of 1.0 μs trajectories using the (b) ff14 DESRES force field starting with the NOE structure (PDB: 1ANR), (c-f) ff99bsc0χOL3 force field starting with (c) the NOE structure (PDB 1ANR), (d) 1ANR with the pucker of U23 changed to C2′-*endo* ($TAR^{U23C2'\text{-}endo}$), (e) 1ANR with the pucker of U25 sugar changed to C2′-*endo* ($TAR^{U25C2'\text{-}endo}$), (f) a FARFAR-NMR conformer in which all bulge nucleotides are C2′-*endo* (TAR^{FARFAR}), and (g) the ff99 force field. The sugar pucker of the starting structures are indicated using a green line. Persistence of a general bias towards C3′-*endo* or a tendency to maintain the sugar pucker in the initial starting conformation can be seen.

Supplementary Discussion

Implication of motional averaging on A-form helix

As expected, very good agreement was observed for both FARFAR and Anton-MD derived ensembles for the central Watson-Crick bps in the two helices (C19-G43, A20-U42, G21-C41, A27-U38, G28-C37), which have more rigid structures and thus should be easier to model. The slightly better agreement observed for C5′, C6 and C1′ chemical shifts for the Anton-MD-NMR as compared to the FARFAR-NMR (Supplementary Fig. 3) is presumably due to deviations from the assumed idealized static A-form geometry and/or motional averaging of the chemical shifts. Single conformers in the Anton-MD-NMR ensemble show slightly weaker agreement (overall RMSD difference < 0.22 ppm) compared to averaging over all conformers in the ensemble, including for C5′, C6 and C1′, suggesting that the better agreement in the case of Anton-MD-NMR is more likely due to neglect of motional averaging for the helices in the FARFAR-NMR ensemble.

Junctional topology dynamics of FARFAR-NMR and Anton-MD-NMR

In the FARFAR-NMR ensemble, the major topology (~75%) of the two-way junction is the canonical trinucleotide U23C24U25 bulge (3:0) (Supplementary Fig. 5a e.g. conformer (5)). However, there is also a minor topology (~25%) in which the trinucleotide bulge migrates one nucleotide down the lower stem to form a AUC bulge (Supplementary Fig. 5a e.g. conformer (10)). In contrast, in the Anton-MD-NMR ensemble for TAR, the topology of the junction varies widely (Supplementary Fig. 5b). The dominant topology (~30%) is the 4:1 internal loop lacking A22-U40 pairing and the U23C24U25 bulge, while the 3:0 internal loop with UCU bulge and a A22-U40 bp is only a minor population (~10%). The absence of base-pairing at A22-U40 is in agreement with the NMR data showing no detectable hydrogen bonds between A22 and U40. The Anton-MD-NMR topologies also include 3:0 or 4:1 internal loops (~30%) in which U25 and U40 form a U25-U40 mismatch with A22-U40 unpaired or both A22-U40 and G21-

C41 unpaired, respectively. In addition, topologies with a 5:2 internal loop (~15%) in which A22-U40 as well as either G21-C41 or G26-C39 are unpaired, and those in which the entire upper helix is melted (~5 %) are also observed. Unpairing in the upper helix and at G21-C41 and G26-C39 is inconsistent with the sharp imino resonances observed for G21 and G26[3]. These differences in pairing may help explain the better predictions of imino $^{15}N/^{1}H$ chemical shifts for the FARFAR-NMR relative to the Anton-MD-NMR ensemble (Fig. 3 and Supplementary Fig. 3). Thus, artifactual distortions in junction topology and helical base pairing in the Anton-MD ensembles appear to compensate for lack of sugar-backbone sampling to achieve the inter-helical orientations needed to satisfy the helical RDC data (Supplementary Fig. 2e-g).

Supplementary Tables

Supplementary Table 1. RDCs datasets used in this study

RNA	Elongation	Apical loop	+/– Mg^{2+}	Number of RDCs	Reference
U1-TAR	E0	wild-type	–	35	[4]
	E0	wild-type	+	38	[4]
U2-TAR	E0	wild-type	–	27	[4]
	EI22	UUCG	–	35	[5]
TAR	E0	UUCG	–	35	[6]
	EI22	UUCG	–	39	[5]
	EII22	UUCG	–	34	[5]
	EI3	UUCG	–	35	[7]
	E0	wild-type	+	46	[4]
U7-TAR	E0	wild-type	–	34	[4]
	E0	wild-type	+	36	[4]
human telomerase P2ab	E0	N/A	–	88	[8]
fluoride riboswitch	E0	N/A	+	89	[9]
preQ1 Class I riboswitch	E0	N/A	–	90	[10]
preQ1 Class II riboswitch	E0	N/A	–	132	[11]

Supplementary Table 2. FARFAR input files and commands (TAR)

2° structure	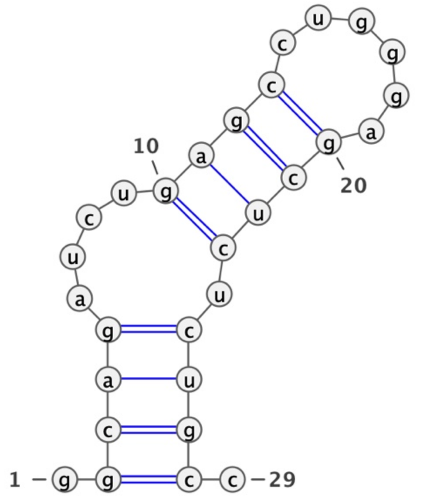
Input files	*cat test.fasta*<br>*> tar*<br>*ggcagaucugagccugggagcucucugcc*<br><br>*cat test.secstruct*<br>*.((((....((((......)))).)))).*<br>*ggcagaucugagccugggagcucucugcc*
Generate RNA helices	*rna_helix.py -seq gcag cugc -resnum 2-5 25-28 -o helix_1.pdb -rosetta_folder ~/rosetta/main/source/cmake/build_release*<br>*rna_helix.py -seq gagc gcuc -resnum 10-13 20-23 -o helix_2.pdb -rosetta_folder ~/rosetta/main/source/cmake/build_release*
FARFAR run	*rna_denovo -nstruct 100 -s helix_*.pdb -secstruct_file test.secstruct -fasta test.fasta -minimize_rna true*

Supplementary Table 3. FARFAR input files and commands (U1-TAR)

2° structure	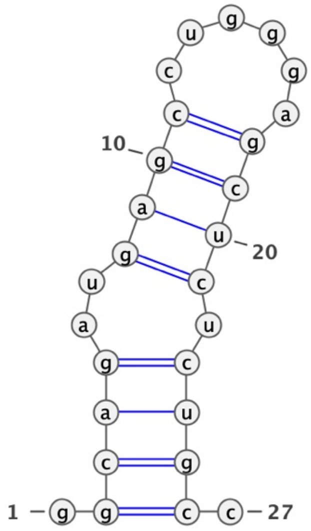
Input files	*cat test.fasta*<br>*> u1-tar*<br>*ggcagaugagccugggagcucucugcc*<br><br>*cat test.secstruct*<br>*.((((..((((......)))).)))).*<br>*ggcagaugagccugggagcucucugcc*
Generate<br><br>RNA helices	*rna_helix.py -seq gcag cugc -resnum 2-5 23-26 -o helix_1.pdb -rosetta_folder ~/rosetta/main/source/cmake/build_release*<br>*rna_helix.py -seq gagc gcuc -resnum 8-11 18-21 -o helix_2.pdb -rosetta_folder ~/rosetta/main/source/cmake/build_release*
FARFAR<br><br>run	*rna_denovo -nstruct 100 -s helix_*.pdb -secstruct_file test.secstruct -fasta test.fasta -minimize_rna true*

Supplementary Table 4. FARFAR input files and commands (U2-TAR)

2° structure	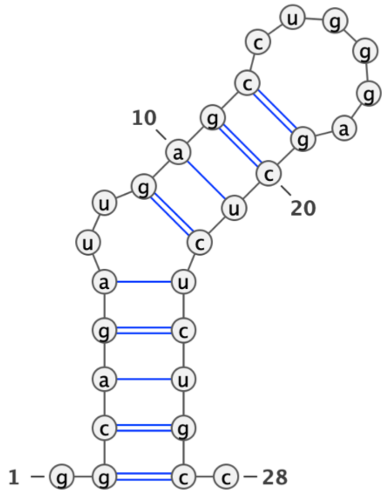
Input files	*cat test.fasta*<br>*> u2-tar*<br>*ggcagauugagccugggagcucucugcc*<br><br>*cat test.secstruct*<br>*.(((((..((((......))))))))).*<br>*ggcagauugagccugggagcucucugcc*
Generate<br><br>RNA helices	*rna_helix.py -seq gcag cugc -resnum 2-5 24-27 -o helix_1.pdb -rosetta_folder ~/Rosetta/main/source/cmake/build_release*<br>*rna_helix.py -seq gagc gcuc -resnum 9-12 19-22 -o helix_2.pdb -rosetta_folder ~/rosetta/main/source/cmake/build_release*
FARFAR<br><br>run	*rna_denovo -nstruct 100 -s helix_*.pdb -secstruct_file test.secstruct -fasta test.fasta -minimize_rna true*

Supplementary Table 5. FARFAR input files and commands (U7-TAR)

2° structure	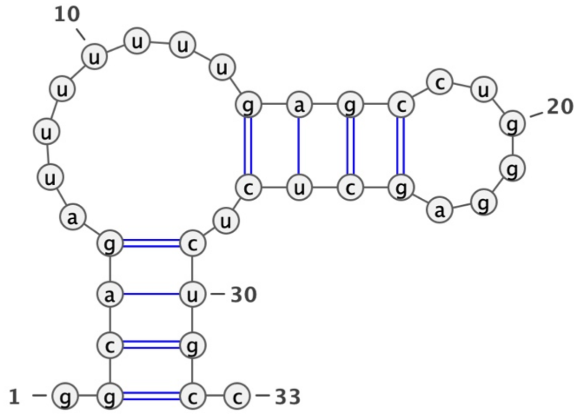
Input files	*cat test.fasta*<br>*> u7-tar*<br>*ggcagauuuuuuugagccugggagcucucugcc*<br><br>*cat test.secstruct*<br>*.((((........((((......)))).)))).*<br>*ggcagauuuuuuugagccugggagcucucugcc*
Generate RNA helices	*rna_helix.py -seq gcag cugc -resnum 2-5 29-32 -o helix_1.pdb -rosetta_folder ~/rosetta/main/source/cmake/build_release*<br>*rna_helix.py -seq gagc gcuc -resnum 14-17 24-27 -o helix_2.pdb -rosetta_folder ~/rosetta/main/source/cmake/build_release*
FARFAR run	*rna_denovo -nstruct 100 -s helix_*.pdb -secstruct_file test.secstruct -fasta test.fasta -minimize_rna true*

Supplementary Table 6. FARFAR input files and commands (Human Telomerase P2ab)

2° structure	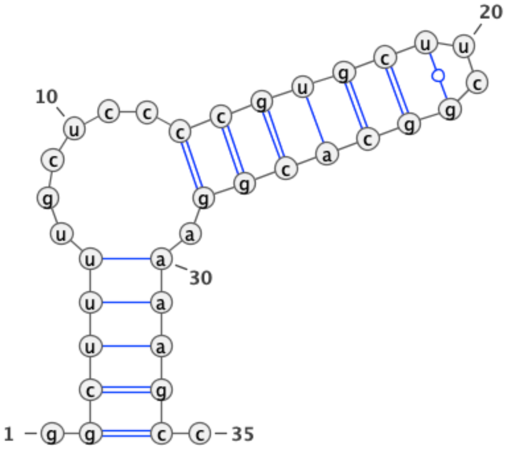
Input files	`cat test.fasta`<br>`> p2ab`<br>`ggcuuuugcuccccgugcuucggcacggaaaagcc`<br><br>`cat test.secstruct`<br>`.(((((......(((((([..])))))).))))).`<br>`ggcuuuugcuccccgugcuucggcacggaaaagcc`
Generate<br><br>RNA helices	`rna_helix.py -seq gcuuuu aaaagc -resnum 2-7 29-34 -o helix_1.pdb -rosetta_folder ~/rosetta/main/source/cmake/build_release`<br>`rna_helix.py -seq ccgugc gcacgg -resnum 13-18 23-28 -o helix_2.pdb -rosetta_folder ~/rosetta/main/source/cmake/build_release`
FARFAR<br><br>run	`rna_denovo -nstruct 100 -obligate_pair_explicit 19 22 S W T -s helix_*.pdb -secstruct_general_file test.secstruct -fasta test.fasta  -minimize_rna true`

Supplementary Table 7. FARFAR input files and commands (Fluoride Riboswitch)

2° structure	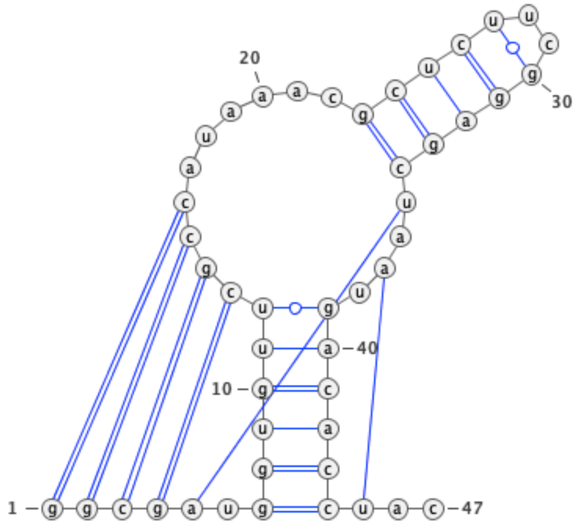
Input files	*cat test.fasta*<br>*> fsw*<br>*ggcgauggguguucgccauaaacgcucuucggagcuaaugacaccuac*<br><br>*cat test.secstruct*<br>*(((([.{{{{{{))))......((((((..))))))].(.}}}}}}).. *<br>*ggcgauggguguucgccauaaacgcucuucggagcuaaugacaccuac*
Generate RNA helices	*rna_helix.py -seq ggcg cgcc -resnum 1-4 13-16 -o helix_1.pdb -rosetta_folder ~/rosetta/main/source/cmake/build_release*<br>*rna_helix.py -seq gguguu gacacc -resnum 7-12 39-44 -o helix_2.pdb -rosetta_folder ~/rosetta/main/source/cmake/build_release*<br>*rna_helix.py -seq gcuc gagc -resnum 23-26 31-34 -o helix_3.pdb -rosetta_folder ~/rosetta/main/source/cmake/build_release*
FARFAR run	*rna_denovo -nstruct 100 -obligate_pair_explicit 27 30 S W T 5 35 W W T 37 45 H W T -s helix_*.pdb -secstruct_general_file test.secstruct -fasta test.fasta -minimize_rna true*

Supplementary Table 8. FARFAR input files and commands (PreQ1 Class I Riboswitch)

2° structure	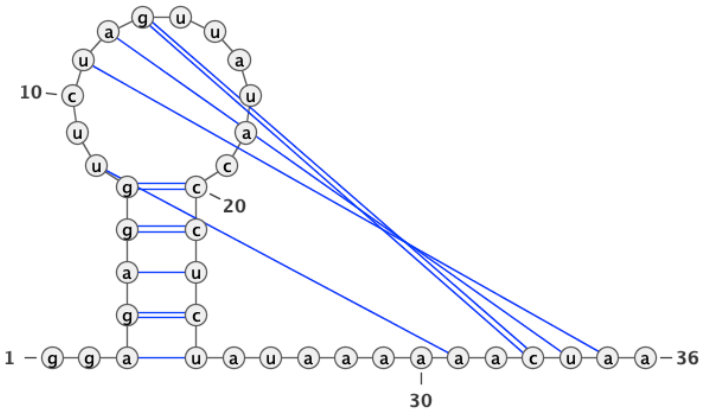
Input files	*cat test.fasta*<br>*> preq1 class I*<br>*ggagagguucuaguuauacccucuauaaaaaacuaa*<br><br>*cat test.secstruct*<br>*..((((([..{{{......)))))......].}}}.*<br>*ggagagguucuaguuauacccucuauaaaaaacuaa*
Generate<br><br>RNA helices	
FARFAR run	*rna_denovo -nstruct 100 -obligate_pair_explicit 4 25 S H T 5 27 S H C 7 30 S W T 8 31 W H C 8 32 W H C 13 18 S W T 20 31 S W C 21 29 S W C -secstruct_general_file test.secstruct -fasta test.fasta -minimize_rna true*

Supplementary Table 9. FARFAR input files and commands (PreQ1 Class II Riboswitch)

2° structure	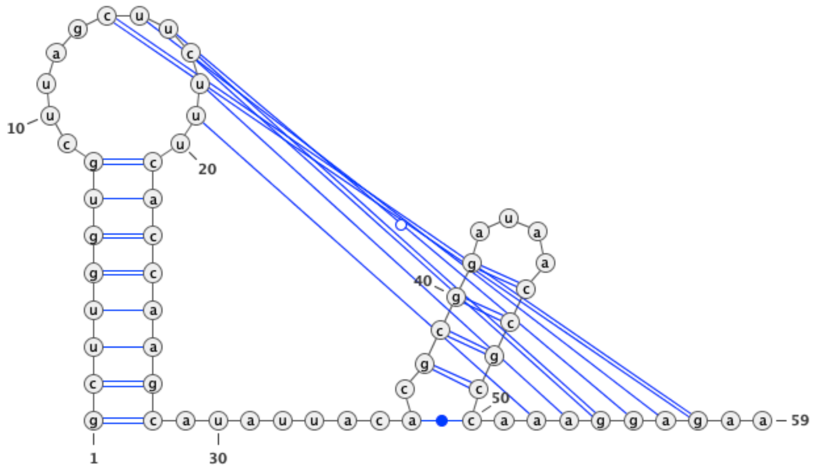
Input files	*cat test.fasta*<br>*> preq1 class II*<br>*gcuuggugcuuagcuucuuucaccaagcauauuacacgcggauaacc*<br>*gccaaaggagaa*<br><br>*cat test.secstruct*<br>*((((((((.....[[[[[[.))))))))).......{.((((....))*<br>*))}.]]]]]]..*<br>*gcuuggugcuuagcuucuuucaccaagcauauuacacgcggauaacc*<br>*gccaaaggagaa*
Generate<br><br>RNA helices	
FARFAR run	*rna_denovo -nstruct 100 -obligate_pair_explicit 10 52 W H C 11 53 W H C 36 50 H W T 36 52 W S T -secstruct_general_file test.secstruct -fasta test.fasta -minimize_rna true*

Reference

1. Salmon, L. et al. Modulating RNA Alignment Using Directional Dynamic Kinks: Application in Determining an Atomic-Resolution Ensemble for a Hairpin using NMR Residual Dipolar Couplings. *Journal of the American Chemical Society* **137**, 12954-12965 (2015).
2. Shi, H. et al. Atomic structures of excited state A–T Hoogsteen base pairs in duplex DNA by combining NMR relaxation dispersion, mutagenesis, and chemical shift calculations. *J. Biomol. NMR* **70**, 229-244 (2018).
3. Dethoff, E.A. et al. Characterizing complex dynamics in the transactivation response element apical loop and motional correlations with the bulge by NMR, molecular dynamics, and mutagenesis. *Biophys J* **95**, 3906-15 (2008).
4. Merriman, D.K. et al. Increasing the length of poly-pyrimidine bulges broadens RNA conformational ensembles with minimal impact on stacking energetics. *RNA* **24**, 1363-1376 (2018).
5. Zhang, Q., Stelzer, A.C., Fisher, C.K. & Al-Hashimi, H.M. Visualizing spatially correlated dynamics that directs RNA conformational transitions. *Nature* **450**, 1263-7 (2007).
6. Al-Hashimi, H.M. et al. Concerted motions in HIV-1 TAR RNA may allow access to bound state conformations: RNA dynamics from NMR residual dipolar couplings. *Journal of Molecular Biology* **315**, 95-102 (2002).
7. Dethoff, E.A., Hansen, A.L., Zhang, Q. & Al-Hashimi, H.M. Variable helix elongation as a tool to modulate RNA alignment and motional couplings. *Journal of Magnetic Resonance* **202**, 117-121 (2010).
8. Zhang, Q., Kim, N.K., Peterson, R.D., Wang, Z. & Feigon, J. Structurally conserved five nucleotide bulge determines the overall topology of the core domain of human telomerase RNA. *Proc Natl Acad Sci U S A* **107**, 18761-8 (2010).
9. Zhao, B., Guffy, S.L., Williams, B. & Zhang, Q. An excited state underlies gene regulation of a transcriptional riboswitch. *Nat. Chem. Biol.* **13**, 968-974 (2017).
10. Zhang, Q., Kang, M., Peterson, R.D. & Feigon, J. Comparison of solution and crystal structures of preQ1 riboswitch reveals calcium-induced changes in conformation and dynamics. *J Am Chem Soc* **133**, 5190-3 (2011).
11. Kang, M., Eichhorn, C.D. & Feigon, J. Structural determinants for ligand capture by a class II preQ1 riboswitch. *Proc Natl Acad Sci U S A* **111**, E663-71 (2014).
