# Supplementary material for: Rapid and accurate determination of atomistic RNA dynamic ensemble models using NMR and structure prediction
Source: Nat Commun. 2020 Nov 2;11:5531. doi: 10.1038/s41467-020-19371-y (PMC7608651; doi:10.1038/s41467-020-19371-y)
Supplement: Supplementary file 4 — Description of Additional Supplementary Files [file 41467_2020_19371_MOESM4_ESM.pdf]

Description of Additional Supplementary Files

Supplementary Movie 1

The FARFAR-NMR atomic-resolution dynamic ensemble of HIV-1 TAR. Flipped out bulge residues are in red, flipped in bulge nucleotides are in blue, and coaxial conformations are in green.
