# Supplementary material for: Rapid and accurate determination of atomistic RNA dynamic ensemble models using NMR and structure prediction
Source: Nat Commun. 2020 Nov 2;11:5531. doi: 10.1038/s41467-020-19371-y (PMC7608651; doi:10.1038/s41467-020-19371-y)
Supplement: Supplementary file 2 — Peer Review File [file 41467_2020_19371_MOESM2_ESM.pdf]

**Editorial Note:** This manuscript has been previously reviewed at another journal that is not operating a transparent peer review scheme. This document only contains reviewer comments and rebuttal letters for versions considered at *Nature Communications.*

Reviewer #1 (Remarks to the Author):

The authors have addressed all questions addressed by the reviewers adequately, and I recommend acceptance as is in Nature Commun
